# Robust synchronization control scheme of a population of nonlinear stochastic synthetic genetic oscillators under intrinsic and extrinsic molecular noise via quorum sensing

**DOI:** 10.1186/1752-0509-6-136

**Published:** 2012-10-26

**Authors:** Bor-Sen Chen, Chih-Yuan Hsu

**Affiliations:** 1 Lab of Control and Systems Biology, Department of Electrical Engineering, National Tsing Hua University, Hsinchu, 30013, Taiwan

## Abstract

**Background:**

Collective rhythms of gene regulatory networks have been a subject of considerable interest for biologists and theoreticians, in particular the synchronization of dynamic cells mediated by intercellular communication. Synchronization of a population of synthetic genetic oscillators is an important design in practical applications, because such a population distributed over different host cells needs to exploit molecular phenomena simultaneously in order to emerge a biological phenomenon. However, this synchronization may be corrupted by intrinsic kinetic parameter fluctuations and extrinsic environmental molecular noise. Therefore, robust synchronization is an important design topic in nonlinear stochastic coupled synthetic genetic oscillators with intrinsic kinetic parameter fluctuations and extrinsic molecular noise.

**Results:**

Initially, the condition for robust synchronization of synthetic genetic oscillators was derived based on Hamilton Jacobi inequality (HJI). We found that if the synchronization robustness can confer enough intrinsic robustness to tolerate intrinsic parameter fluctuation and extrinsic robustness to filter the environmental noise, then robust synchronization of coupled synthetic genetic oscillators is guaranteed. If the synchronization robustness of a population of nonlinear stochastic coupled synthetic genetic oscillators distributed over different host cells could not be maintained, then robust synchronization could be enhanced by external control input through quorum sensing molecules. In order to simplify the analysis and design of robust synchronization of nonlinear stochastic synthetic genetic oscillators, the fuzzy interpolation method was employed to interpolate several local linear stochastic coupled systems to approximate the nonlinear stochastic coupled system so that the HJI-based synchronization design problem could be replaced by a simple linear matrix inequality (LMI)-based design problem, which could be solved with the help of LMI toolbox in MATLAB easily.

**Conclusion:**

If the synchronization robustness criterion, i.e. the synchronization robustness ≥ intrinsic robustness + extrinsic robustness, then the stochastic coupled synthetic oscillators can be robustly synchronized in spite of intrinsic parameter fluctuation and extrinsic noise. If the synchronization robustness criterion is violated, external control scheme by adding inducer can be designed to improve synchronization robustness of coupled synthetic genetic oscillators. The investigated robust synchronization criteria and proposed external control method are useful for a population of coupled synthetic networks with emergent synchronization behavior, especially for multi-cellular, engineered networks.

## Background

Many biochemically dynamical systems are controlled by intrinsic rhythms generated by specialized cellular clocks within the organism itself. These rhythm generators are composed of thousands of clock cells that are intrinsically diverse, but nevertheless manage to function in a coherent oscillatory state [1-6]. However, the synchronization mechanisms by which this collective behavior arises remains to be understood, even if individual clock cells are known to operate through biochemical networks comprising of multiple regulatory feedback loops [7-11]. The complexity of these cellular systems has hindered a complete understanding of natural genetic oscillators and their synchronization [12-16].

Recently designed synthetic genetic oscillators can offer an alternative approach, and provide a relatively well-controlled test bed in which the function and behavior of natural genetic oscillators can be isolated and characterized in detail [1,3,7,8,17]. As an example, a synthetic biological oscillator, termed the “repressilator,” was developed in *Escherichia coli* from a network of three transcriptional repressors that inhibit one another in a cyclic way [18-21]. Spontaneous oscillations were observed in individual cells within a growing culture, although substantial variability and noise were present among the different cells. Another synthetic oscillator was designed and built that exhibited damped oscillatory responses to perturbations in culture [22]. Recently, several mechanisms of intercell coupling of synthetic genetic oscillators have been discussed, to enhance the oscillating response of the synthetic biological system [1]. In general, coupling among oscillators is not sufficient to achieve synchronization, and many ensembles of coupled oscillators exhibit phase dispersion rather than a synchronized state, because the oscillators may actively resist oscillation or because the coupling is too small. Therefore, the synchronization of a population of nonlinear stochastic coupled oscillators must be analyzed carefully.

In previous studies [1,12-16,23-26], the collective behavior of synthetic genetic oscillators was discussed on the basis of cell-to-cell communication through quorum sensing, which may lead to synchronization in an ensemble of identical genetic oscillators. In general, intercellular communication is accomplished by transmitting individual cell reactions via intercellular signals to neighboring cells, which can generate a global cellular synchronization at the level of molecules, tissues, organs, or the body [1]. The ability to communicate among cells is an absolute requisite to ensure appropriate and robust synchronization at all levels in organisms living in uncertain environments. Synchronization of coupled networks has been investigated intensively in past decades because of its biological implications and potential applications [3,4,20]. The global synchronization mechanism of oscillators via direct coupling has been derived based on the Lyapunov method and linearization technique [27-29]. Different synchronization mechanisms in a population of nonlinear stochastic genetic oscillators with noise and impulse control inputs have also been previously discussed [7-9].

Generally, biological systems or organisms are subject to time-varying uncertainties, assumed to be in the form of both internal noise resulting from the birth and death of biochemical molecules, and external noise resulting from environmental perturbations [15,19,30-34]. It has been shown that environmental molecular noise plays an important role in the stochastic behavioral phenomena of biological systems at various levels. In particular, gene regulation is an inherently noisy process [35-37], from transcriptional control, alternative splicing, translation, and diffusion to biological modification of transcription factors. Such stochastic noise cannot only significantly affect the dynamics of biological systems, but also may be exploited by living organisms to actively facilitate certain cellular functions, such as cellular communication and synchronization. In this study, the time-varying parameter fluctuations in gene regulation processes are modeled as stochastic intrinsic kinetic noise, whereas environmental molecular noise of the host cells is modeled as an extrinsic disturbance of synthetic genetic oscillators. A population of synthetic oscillators coupled by quorum sensing is modeled by a set of coupled nonlinear stochastic equations with intrinsic and extrinsic noise. Our main purpose is to discuss the robust synchronization mechanism of a population of nonlinear stochastic coupled synthetic genetic oscillators under intrinsic kinetic parameter fluctuations and extrinsic environmental molecular noise on the host cells.

Based on nonlinear stochastic equations of coupled synthetic genetic oscillators distributed in different host cells, the robust synchronization mechanism is discussed from the *H*_*∞*_ noise-filtering perspective. The robust ability to tolerate stochastic kinetic parameter fluctuations and the filtering ability to attenuate extrinsic environmental molecular noise to maintain synchronization of nonlinear stochastic coupled synthetic genetic oscillations was measured from the nonlinear stochastic system theory point of view. In the case where robust synchronization cannot be maintained or is corrupted by intrinsic kinetic parameter fluctuations and extrinsic environmental molecular noise, some robust synchronization design methods are discussed to enhance synchronization. Both the physical insight into the robust synchronization mechanisms and the designs to improve these mechanisms require solving nonlinear HJIs, which cannot be easily achieved by any analytical or numerical method at present. In order to simplify this analysis and design, the Takagi-Sugeno (T-S) fuzzy model [38-40] is employed to interpolate several local linear stochastic genetic oscillators to approximate the nonlinear stochastic genetic oscillators. Consequently, the analysis and design of robust synchronization can be achieved by solving of a set of LMIs [41], via the help of the LMI toolbox in MATLAB.

If the robust synchronization of nonlinear stochastic coupled synthetic genetic oscillators could not be achieved spontaneously, an external control input was developed to synchronize the coupled synthetic genetic oscillators. External stimulation inputs are known to play an important role in the synchronization of biological rhythms. For instance, many organisms display a circadian rhythm of 24-hours periodicity entrained to the light–dark cycle [42]. Other examples include physiological rhythms stimulated by regular or periodic inputs occurring in the context of medical devices, synchronization of electronic genetic networks by an external forcing, i.e., external voltage [43], and a wide variety of regular and irregular rhythms induced by periodic stimulation of squid giant axons [21]. In general, physiological oscillations can be synchronized by appropriate external or internal stimuli. Recently, Wang *et al*. constructed an impulse control system to model the process of periodical injection of coupling substances with constant or random impulse control into a common extracellular medium and studied its effect on the dynamics of collective rhythms [9]. Synchronization with time delays via direct coupling and linearization methods has also been discussed [29,43]. In our study, a control input design method was developed to guarantee the robust synchronization of synthetic genetic oscillators under intrinsic kinetic parameter fluctuations and extrinsic environmental molecular noise. In order to avoid solving the complicated HJI, the fuzzy interpolation method was also employed to simplify the control design procedure by only solving a set of simple LMIs [41].

The contributions of this paper are fourfold: (1) A nonlinear stochastic system is introduced to model a population of nonlinear stochastic coupled synthetic genetic oscillators under random intrinsic kinetic parameter fluctuations and extrinsic molecule noise *in vivo*; (2) The conditions of robust synchronization are developed from the nonlinear stochastic dynamical system point of view, i.e. if the synchronization robustness ≥ intrinsic robustness + extrinsic robustness, then the intrinsic parameter fluctuation can be tolerated and the extrinsic noise can be buffered so that the robust synchronization of coupled oscillators can be guaranteed. Therefore, we could obtain better insight into synchronization mechanisms of coupled synthetic molecular systems distributed in host cells, and to provide further systematic analysis and control design to improve the synchronization robustness. Further, if the robust synchronization conditions cannot be guaranteed, some synchronization control schemes are also developed to improve the synchronization robustness of coupled synthetic gene networks; (3) The fuzzy interpolation method is introduced to simplify the analysis and design procedure of robust synchronization of coupled nonlinear stochastic synthetic gene networks; and (4) An external inducer input control design method is also developed to guarantee robust synchronization of the nonlinear stochastic synthetic genetic oscillators, when spontaneous synchronization cannot be achieved under intrinsic kinetic parameter fluctuations and extrinsic environmental molecular noise. Finally, a design example is provided *in silico* to illustrate the design procedure and to confirm the performance of the proposed design methods.

## Methods

### Stochastic models of nonlinear stochastic synthetic genetic oscillators under intrinsic kinetic parameter fluctuations and external molecular noise

#### Model description

**Figure 1:**
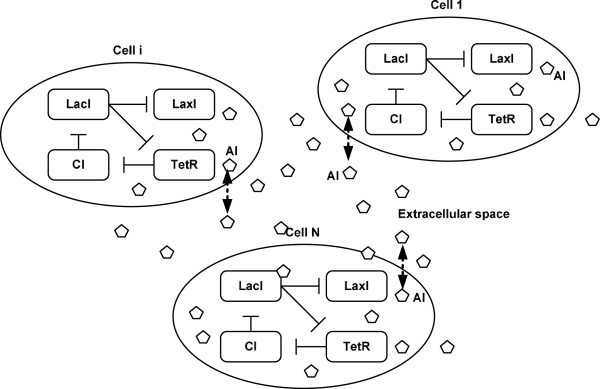
**Synchronization scheme of *N *coupled genetic oscillators distributed in different host cells by the quorum sensing mechanism.**

Before discussion of synchronization of more general synthetic genetic oscillators, we here provide a design example of coupled repressilators to illustrate the interesting phenomenon of synchronization in coupled dynamic cells. Then model description, definition, and theoretical results of robust synchronization of more general coupled synthetic genetic networks will be introduced for further study in the sequel. The repressilator is a network of three genes, the products of which inhibit the transcription of each other in a cyclical manner [1]. The gene *lacI* (from *E. coli*.) codes for the protein LacI, which inhibits the transcription of the gene *tetR*. The product of the latter, TetR, inhibits the transcription of gene *cI* (from λ phage); the protein product CI in turn inhibits the expression of *lacI*, thus completing the cycle. Garcia-Ojalvo *et al*. proposed a modular addition to the repressilator, with the aim of coupling a population of cells containing this network [1]. The basic mechanism of communication among the cells is based on quorum sensing, which was first discovered in the bioluminescent bacteria, *Vibrio fisceri*. These bacteria exhibit collective behaviors, mainly using two proteins (see Figure 1). The first protein, LuxI, synthesizes a small molecule known as an auto-inducer (AI), which diffuses freely through the cell membrane. The second protein, LuxR, binds to the AI molecule to form a complex, which subsequently activates transcription of various genes, as shown in Figure 1 (a detailed description of this molecular pathway is provided by Garcia-Ojalvo *et al*. [1]). Recently, several studies have indicated that quorum sensing can be engineered and used as an intercellular signaling system in *E. coli*[1,7,9,15-17].

Based on the synchronization scheme of coupled repressilators via quorum sensing mechanism (Figure 1), the mRNA dynamics in the cell *i* = 1, 2, …, *N* are governed by repressible transcription of all three genes of the repressilator plus transcription activation of the additional copy of the *lacI* gene, and by mRNA degradation [7].

(1) $$\begin{array}{l} \frac{dx_{\mathrm{ai}}\left(t\right)}{dt}=-\gamma_{m}x_{\mathrm{ai}}\left(t\right)+\frac{\alpha_{a}}{\mu +x_{\mathrm{Ci}}^{n}\left(t\right)}\\ \frac{dx_{\mathrm{bi}}\left(t\right)}{dt}=-\gamma_{m}x_{\mathrm{bi}}\left(t\right)+\frac{\alpha_{b}}{\mu +x_{\mathrm{Ai}}^{n}\left(t\right)}\;,i=1,2,\ldots ,N\\ \frac{dx_{\mathrm{ci}}\left(t\right)}{dt}=-\gamma_{m}x_{\mathrm{ci}}(t)+\frac{\alpha_{c}}{\mu +x_{\mathrm{Bi}}^{n}\left(t\right)}\\ \;+\frac{\alpha_{S}x_{\mathrm{Si}}\left(t\right)}{\mu_{S}+x_{\mathrm{Si}}\left(t\right)} \end{array}$$

where *x*_*ai*_ , *x*_*bi*_ and *x*_*ci*_ are the concentration of mRNA transcribed from *tetR*, *cI* and *lacI* in cell *i*, respectively; concentrations of the corresponding proteins are represented by *x*_*Ai*_ , *x*_*Bi*_ and *x*_*Ci*_ , respectively. The concentration of AI inside each cell is denoted by *x*_*Si*_. *α*_*a*_, *α*_*b*_, and *α*_*c*_ are the dimensionless transcription rate in the absence of the repressor, and *μ* is the repression coefficient. *α*_*S*_ is the maximal contribution to *lacI* transcription in the presence of saturating amounts of AI, and *μ*_*S*_ is the activation coefficient. *γ*_*m*_ is the respective dimensionless degradation rate of mRNA for *tetR*, *cI* and *lacI* in the cell, and *n* is the Hill coefficient [7].

The dynamics of proteins TetR, CI and LacI are given respectively as [7]

(2) $$\begin{array}{l} \frac{dx_{\mathrm{Ai}}\left(t\right)}{dt}=-\gamma_{p}x_{\mathrm{Ai}}\left(t\right)+\beta_{A}x_{\mathrm{ai}}\left(t\right)\\ \frac{dx_{\mathrm{Bi}}\left(t\right)}{dt}=-\gamma_{p}x_{\mathrm{Bi}}\left(t\right)+\beta_{B}x_{\mathrm{bi}}\left(t\right)\;,i=1,2,\ldots ,N\\ \frac{dx_{\mathrm{Ci}}\left(t\right)}{dt}=-\gamma_{p}x_{\mathrm{Ci}}\left(t\right)+\beta_{C}x_{\mathrm{ci}}\left(t\right) \end{array}$$

where parameters *β*_*A*_*, β*_*B*_ and *β*_*C*_ are the translation rates of the proteins from their mRNA, and *γ*_*P*_ represents the dimensionless degradation rate of proteins TetR, CI, and LacI in the cell. The intercellular protein AI in cell *i* is synthesized by the protein LuxI and diffuses through the cell wall where it undergoes degradation, leading to the following equation [7].

(3) $$\begin{array}{c} \frac{dx_{\mathrm{Si}}\left(t\right)}{dt}=-\gamma_{s}x_{\mathrm{Si}}\left(t\right)+\beta_{s}x_{\mathrm{Ai}}\left(t\right)\\ \;-\eta_{s}Q_{e}\left(x_{\mathrm{Si}}\left(t\right)-N^{-1}\sum_{j=1}^{N}x_{\mathrm{Sj}}\left(t\right)\right),\;0\leq Q_{e}\leq 1 \end{array}$$

where *η*_s_ measures the diffusion rate of AI across the cell membrane, *β*_*s*_ is the synthesis rate of AI, and *γ*_*s*_ gives the rate of decay of AI. Consequently, the whole coupled synthetic system is expressed by (1)-(3), which can be represented by the following more generalized nonlinear dynamic equation

(4) $${\dot{x}}_{i}\left(t\right)=f\left(x_{i}\left(t\right)\right)+\sum_{j=1}^{N}c_{\mathrm{ij}}g\left(x_{j}\left(t\right)\right),\;i=1,2,\ldots ,N$$

where *x*_*i*_(*t*) = (*x*_*ai*_(*t*), *x*_*bi*_(*t*), *x*_*ci*_(*t*), *x*_*Ai*_(*t*), *x*_*Bi*_(*t*), *x*_*Ci*_(*t*), *x*_*Si*_(*t*))^*T*^ ∈ *R*^*m*^ is the state vector of the *i*th synthetic oscillator; *f*(·) : *R*^*m*^ → *R*^*m*^ is a smooth nonlinear function that characterizes the behavior of the synthetic oscillator; *g*(·) : *R*^*m*^ → *R*^*m*^ is a smooth nonlinear inner-coupling function; and *C* = (*c*_*ij*_)_*N* × *N*_ ∈ *R*^*N* × *N*^ is the coupling configuration matrix, where *c*_*ij*_*>0* means that the *i*th synthetic oscillator is coupled with the *j*th synthetic oscillator directly, otherwise *c*_*ij*_=0. Assume that the diagonal elements of *C* satisfy $c_{\mathrm{ii}}=-\sum_{j=1,j\neq i}^{N}c_{\mathrm{ij}}$.

#### Synthetic genetic oscillators under intrinsic parameter fluctuations

In general, the synthetic genetic oscillators suffer kinetic parameter fluctuations from transcription control, alternative splicing, translation, genetic mutation and diffusion to biological modification of transcription factors [17,19,31,34,44]. In this situation, dynamic equations of coupled synthetic genetic oscillators in (1)-(3) are modified as

(5) $$\begin{array}{l} \frac{dx_{\mathrm{ai}}\left(t\right)}{dt}=-\left(\gamma_{m}+\Delta \gamma_{m}n_{i}\left(t\right)\right)x_{\mathrm{ai}}(t)+\frac{\left(\alpha_{a}+\Delta \alpha_{a}n_{i}\left(t\right)\right)}{\mu +x_{\mathrm{Ci}}^{n}\left(t\right)}\\ \frac{dx_{\mathrm{bi}}\left(t\right)}{dt}=-\left(\gamma_{m}+\Delta \gamma_{m}n_{i}\left(t\right)\right)x_{\mathrm{bi}}(t)+\frac{\left(\alpha_{b}+\Delta \alpha_{b}n_{i}\left(t\right)\right)}{\mu +x_{\mathrm{Ai}}^{n}\left(t\right)}\\ \frac{dx_{\mathrm{ci}}\left(t\right)}{dt}=-\left(\gamma_{m}+\Delta \gamma_{m}n_{i}\left(t\right)\right)x_{\mathrm{ci}}(t)+\frac{\left(\alpha_{c}+\Delta \alpha_{c}n_{i}\left(t\right)\right)}{\mu +x_{\mathrm{Bi}}^{n}\left(t\right)}+\frac{\left(\alpha_{S}+\Delta \alpha_{S}n_{i}\left(t\right)\right)x_{\mathrm{Si}}\left(\text{t}\right)}{\mu_{S}+x_{\mathrm{Si}}\left(t\right)}\\ \frac{dx_{\mathrm{Ai}}\left(t\right)}{dt}=-\left(\gamma_{p}+\Delta \gamma_{p}n_{i}\left(t\right)\right)x_{\mathrm{Ai}}(t)+\left(\beta_{A}+\Delta \beta_{A}n_{i}\left(t\right)\right)x_{\mathrm{ai}}(t)\\ \frac{dx_{\mathrm{Bi}}\left(t\right)}{dt}=-\left(\gamma_{p}+\Delta \gamma_{p}n_{i}\left(t\right)\right)x_{\mathrm{Bi}}(t)+\left(\beta_{B}+\Delta \beta_{B}n_{i}\left(t\right)\right)x_{\mathrm{bi}}(t)\\ \frac{dx_{\mathrm{Ci}}\left(t\right)}{dt}=-\left(\gamma_{p}+\Delta \gamma_{p}n_{i}\left(t\right)\right)x_{\mathrm{Ci}}(t)+\left(\beta_{C}+\Delta \beta_{C}n_{i}\left(t\right)\right)x_{\mathrm{ci}}(t)\\ \frac{dx_{\mathrm{Si}}\left(t\right)}{dt}=-\left(\gamma_{s}+\Delta \gamma_{s}n_{i}\left(t\right)\right)x_{\mathrm{Si}}(t)+\left(\beta_{s}+\Delta \beta_{s}n_{i}\left(t\right)\right)x_{\mathrm{Ai}}(t)-\left(\eta_{s}+\Delta \eta_{s}n_{i}\left(t\right)\right)Q_{e}\left(x_{\mathrm{Si}}\left(t\right)-N^{-1}\sum_{j=1}^{N}x_{\mathrm{Sj}}\left(t\right)\right) \end{array}$$

where *Δα*_*j*_, *j* ∈ {*a*, *b*, *c*, *S*}, *Δβ*_*j*_, *j* ∈ {*A*, *B*, *C*, *s*}, *Δγ*_*j*_, *j* ∈ {*m*, *p*, *s*} and Δ*η*_*s*_ denote the amplitudes of the kinetic parameter fluctuations and *n*_*i*_(*t*) is random white noise with zero-mean and unit variance, i.e., Δ*α*_*j*_, Δ*β*_*j*_, Δ*γ*_*j*_ and Δ*η*_*s*_ denote the deterministic part of the stochastic kinetic parameter fluctuations Δ*α*_*j*_*n*_*i*_(*t*), Δ*β*_*j*_*n*_*i*_(*t*), Δ*γ*_*j*_*n*_*i*_(*t*) and *Δη*_*s*_*n*_*i*_(*t*), respectively, and *n*_*i*_(*t*) absorbs the stochastic property of random intrinsic kinetic parameter fluctuations. The covariances of the stochastic parameter fluctuations *Δα*_*j*_*n*_*i*_(*t*), *Δβ*_*j*_*n*_*i*_(*t*), *Δγ*_*j*_*n*_*i*_(*t*) and *Δη*_*s*_*n*_*i*_(*t*) are given as *cov*(*ξn*_*i*_(*t*), *ξn*_*i*_(*t*)) = *ξ*^2^*Δ*_*t*,*τ*_, with *ξ* ∈ {*Δα*_*j*_, *Δβ*_*j*_, *Δγ*_*j*_, *Δη*_*s*_}, respectively, where *Δ*_*t*,*τ*_ denotes the delta function, that is, *Δ*_*t*,*τ*_ = 1 if *t* = *τ* and *Δ*_*t*,*τ*_ = 0 if *t* ≠ *τ*, i.e., *Δα*_*j*_, *Δβ*_*j*_, *Δγ*_*j*_ and *Δη*_*s*_ denote the corresponding standard deviations of the stochastic parameter fluctuations *Δα*_*j*_*n*_*i*_(*t*), *Δβ*_*j*_*n*_*i*_(*t*), *Δγ*_*j*_*n*_*i*_(*t*) and *Δη*_*s*_*n*_*i*_(*t*), respectively. Thus, the nonlinear stochastic coupled synthetic genetic oscillators under intrinsic kinetic parameter fluctuations in the host cell *i* can be represented by

(6) $$\begin{array}{c} {\dot{x}}_{i}\left(t\right)=\left(f\left(x_{i}\left(t\right)\right)+\sum_{j=1}^{N}c_{\mathrm{ij}}g\left(x_{j}\left(t\right)\right)\right)\\ \;+\left(f_{W}\left(x_{i}\left(t\right)\right)+\sum_{j=1}^{N}c_{\mathrm{ij}}g_{W}\left(x_{j}\left(t\right)\right)\right)n_{i}\left(t\right) \end{array}$$

where $\left(f_{W}\left(x_{i}\left(t\right)\right)+\sum_{j=1}^{N}c_{\mathrm{ij}}g_{W}\left(x_{j}\left(t\right)\right)\right)n_{i}\left(t\right)$ denotes the intrinsic kinetic parameter fluctuations of synthetic genetic oscillator in the host cell *i*. For the convenience of analysis and control design of the synchronization in synthetic genetic oscillators inserted into different host cells, the nonlinear stochastic equation in (6) can be represented by the following Ito’s stochastic differential equation

(7) $$\begin{array}{c} dx_{i}\left(t\right)=\left(f\left(x_{i}\left(t\right)\right)+\sum_{j=1}^{N}c_{\mathrm{ij}}g\left(x_{j}\left(t\right)\right)\right)dt\\ \;+\left(f_{W}\left(x_{i}\left(t\right)\right)+\sum_{j=1}^{N}c_{\mathrm{ij}}g_{W}\left(x_{j}\left(t\right)\right)\right)dw_{i}\left(t\right) \end{array}$$

where *w*_*i*_(*t*) is a standard Wiener process or Brownian motion with *dw*_*i*_(*t*) = *n*_*i*_(*t*)*dt* to represent the random kinetic fluctuations of synthetic gene circuits.

#### Synthetic genetic oscillators under external environmental molecular noise

In general, a synthetic genetic oscillator *in vivo* also suffers from extrinsic environmental molecular noise, such as transmitted noise from upstream and global noise affecting all cells. Therefore, the nonlinear stochastic equation of coupled oscillators in (7) should be modified to mimic realistic dynamic behavior, as follows:

(8) $$\begin{array}{c} dx_{i}\left(t\right)=\left(f\left(x_{i}\left(t\right)\right)+\sum_{j=1}^{N}c_{\mathrm{ij}}g\left(x_{j}\left(t\right)\right)+h_{i}v_{i}\left(t\right)\right)dt\\ \;+\left(f_{W}\left(x_{i}\left(t\right)\right)+\sum_{j=1}^{N}c_{\mathrm{ij}}g_{W}\left(x_{j}\left(t\right)\right)\right)dw_{i}\left(t\right) \end{array}$$

where the signal vector *v*_*i*_(*t*) denotes extrinsic environmental molecular noise from the environment and *h*_*i*_ denotes the noise-coupling matrix in cell *i*.

### Robust synchronization control design for nonlinear synthetic genetic oscillators under intrinsic kinetic parameter fluctuations and external environmental molecular noise

The nonlinear stochastic coupled synthetic genetic oscillators in (8) are said to reach synchronization asymptotically if *x*_1_(*t*) = *x*_2_(*t*) = ⋯ = *x*_*N*_(*t*) = *s*(*t*) in probability as *t* → *t*_*f*_ or

(9) $$E\left(x_{i}\left(t\right)-s\left(t\right)\right)=0\;as\;t\to t_{f}$$

in which *s*(*t*) ∈ *R*^*m*^ is the synchronization solution satisfying

(10) $$\begin{array}{c} ds\left(t\right)=\left(f\left(s\left(t\right)\right)+\sum_{j=1}^{N}c_{\mathrm{ij}}g\left(s\left(t\right)\right)+h_{s}v_{i}\left(t\right)\right)dt\\ \;+\left(f_{W}\left(s\left(t\right)\right)+\sum_{j=1}^{N}c_{\mathrm{ij}}g_{W}\left(s\left(t\right)\right)\right)dw_{i}\left(t\right) \end{array}$$

Let us denote the synchronization error signal for synthetic genetic oscillators as

(11) $$e_{i}\left(t\right)=x_{i}\left(t\right)-s\left(t\right),\;i=1,2,\ldots ,N$$

According to (8), the synchronization error dynamics for cell *i* are then described by

(12) $$\begin{array}{c} de_{i}(t)=\left(f\left(x_{i}\left(t\right)\right)-f\left(s\left(t\right)\right)+\sum_{j=1}^{N}c_{\mathrm{ij}}\left(g\left(x_{j}\left(t\right)\right)-g\left(s\left(t\right)\right)\right)+\left(h_{i}-h_{s}\right)v_{i}\left(t\right)\right)dt\\ \;+\left(f_{W}\left(x_{i}\left(t\right)\right)-f_{W}\left(s\left(t\right)\right)+\sum_{j=1}^{N}c_{\mathrm{ij}}\left(g_{W}\left(x_{j}\left(t\right)\right)-g_{W}\left(s\left(t\right)\right)\right)\right)dw_{i}(t) \end{array}$$

for *i* = 1, 2, …, *N* which can be augmented as

(13) $$\begin{array}{c} de=\left(F\left(x,s\right)+\left(C⊗I_{m}\right)G\left(x,s\right)+Hv\right)dt+\left(F_{W}\left(x,s\right)\right)\\ \;+\left(C⊗I_{m}\right)G_{W}\left(x,s\right)dw \end{array}$$

where

$$\begin{array}{c} x=\left(\begin{array}{c} x_{1}\\ \vdots \\ x_{N} \end{array}\right),e=\left(\begin{array}{c} e_{1}\\ \vdots \\ e_{N} \end{array}\right),v=\left(\begin{array}{c} v_{1}\\ \vdots \\ v_{N} \end{array}\right),\\ w=\left(\begin{array}{c} w_{1}\\ \vdots \\ w_{N} \end{array}\right),H=\left(\begin{array}{cc} h_{1}-h_{s} & 0\\ \ddots \\ 0 & h_{N}-h_{s} \end{array}\right)\text{,} \end{array}$$

and

$$\begin{array}{l} F(x,s)=\left(\begin{array}{c} f\left(x_{1}\left(t\right)\right)-f\left(s\left(t\right)\right)\\ \vdots \\ f\left(x_{N}\left(t\right)\right)-f\left(s\left(t\right)\right) \end{array}\right),G(x,s)=\left(\begin{array}{c} g\left(x_{1}\left(t\right)\right)-g\left(s\left(t\right)\right)\\ \vdots \\ g\left(x_{N}\left(t\right)\right)-g\left(s\left(t\right)\right) \end{array}\right),\\ F_{W}(x,s)=\left(\begin{array}{cc} f_{W}\left(x_{1}\left(t\right)\right)-f_{W}\left(s\left(t\right)\right) & 0\\ \ddots \\ 0 & f_{W}\left(x_{N}\left(t\right)\right)-f_{W}\left(s\left(t\right)\right) \end{array}\right),\\ G_{W}(x,s)=\left(\begin{array}{cc} g_{W}\left(x_{1}\left(t\right)\right)-g_{W}\left(s\left(t\right)\right) & 0\\ \ddots \\ 0 & g_{W}\left(x_{N}\left(t\right)\right)-g_{W}\left(s\left(t\right)\right) \end{array}\right) \end{array}$$

Suppose the influence of extrinsic environmental molecular noise on the synchronization error can be bounded by the following noise-filtering level *ρ* of coupled synthetic oscillators [30,31,44]

(14) $$\begin{array}{c} \frac{E\int_{0}^{t_{f}}e^{T}\left(t\right)Re\left(t\right)dt}{E\int_{0}^{t_{f}}v^{T}\left(t\right)v\left(t\right)dt}\leq \rho^{2}\\ or\\ E\int_{0}^{t_{f}}e{\left(t\right)}^{T}Re\left(t\right)dt\leq \rho^{2}E\int_{0}^{t_{f}}v{\left(t\right)}^{T}v\left(t\right)dt \end{array}$$

for all possible environmental noises *v*(*t*), where *ρ*^2^ denotes the upper bound of the effect of *v*(*t*) on synchronization error from the mean energy point of view, i.e., the noise filtering level *ρ* in (14) denotes the upper bound of the noise-filtering ability *ρ*_0_ of coupled synthetic genetic oscillators, and *R* is a symmetric weighting matrix to be specified by designer.

#### Remark 1

(i) The inequality in (14) means that the effect of extrinsic environmental molecular noise on the synchronization error is less than *ρ* from the mean energy point of view, i.e., the value of the noise-filtering ability *ρ*_0_ is the lower bound of *ρ*. Because the statistics of extrinsic molecular noise may be unavailable or uncertain, it is very difficult to obtain the noise filtering ability *ρ*_0_ for all possible extrinsic noises *v*(*t*) directly and only the upper bound *ρ* of the noise-filtering ability *ρ*_0_ can be given in (14) at first. Then, we will decrease the upper bound *ρ* to as small a value as possible to approach its lower bound for the noise-filtering ability *ρ*_0_, i.e., to get *ρ*_0_ by minimizing *ρ* (or *ρ*_0_ = min *ρ*) indirectly. If the noise filtering ability *ρ*_0_ is small, it means that the environmental molecular noise has less influence on synchronization and vice versa. It will be further discussed in the sequel.

(ii) If the extrinsic environmental molecular noise *v* is deterministic, then the expectation on *v*(*t*) in (14) should be neglected. If the initial condition *e*(0) is considered, then the noise-filtering level in (14) should be modified as follows [15,16]:

(15) $$E\int_{0}^{t_{f}}e{\left(t\right)}^{T}Re\left(t\right)dt\leq EV\left(e\left(0\right)\right)+\rho^{2}E\int_{0}^{t_{f}}v{\left(t\right)}^{T}v\left(t\right)dt$$

For some Lyapunov function *V*(*e*(0)), i.e., the energy due to the initial condition *e*(0) should be considered in the influence of noise on synchronization [30,45].

## Results

Based on the synchronization error dynamic equation in (13) and the *H*_∞_ noise filtering performance in (14) or (15), we obtain the following robust synchronization result for nonlinear stochastic coupled synthetic genetic oscillators under intrinsic kinetic parameter fluctuations and extrinsic environmental molecular noise.

### *Proposition 1*

If there exists a positive function *V*(*e*) > 0 with *V*(0) > 0 solving the following HJI,

(16) $$\begin{array}{c} e^{T}Re+{\left(\frac{\partial V\left(e\right)}{\partial e}\right)}^{T}\left(F\left(x,s\right)+\left(C⊗I_{m}\right)G\left(x,s\right)\right)\\ \;+\frac{1}{4\rho^{2}}{\left(\frac{\partial V\left(e\right)}{\partial e}\right)}^{T}HH^{T}\left(\frac{\partial V\left(e\right)}{\partial e}\right)+\frac{1}{2}\left(F_{W}\left(x,s\right)\right)\\ \;+\left(C⊗I_{m}\right)G_{W}\left(x,s\right)\frac{\partial^{2}V\left(e\right)}{\partial e^{2}}\left(F_{W}\left(x,s\right)\right)\\ \;+\left(C⊗I_{m}\right)G_{W}\left(x,s\right)<0 \end{array}$$

then the stochastic intrinsic noise can be tolerated (i.e. the synchronization of coupled synthetic genetic networks cannot destroyed by intrinsic noise) and the influence of extrinsic environmental molecular noise *v*(*t*) on the synchronization of the nonlinear stochastic coupled synthetic oscillation systems in (8) is less than or equal to a prescribed filtering level *ρ*, i.e., the inequality in (14) holds.

#### Proof: see Additional file 1: Appendix A

Since *ρ* denotes an upper bound of the effect of *v*(*t*) on synchronization, the real effect can be obtained by minimizing *ρ* to as small a value as possible. Therefore, the noise-filtering ability of synchronized oscillators on *v*(*t*) can be obtained by solving the following constrained optimization:

(17) $$\rho_{0}=\min\rho$$

subject to HJI in (16) with *V*(*e*) > 0

i.e., the noise-filtering ability *ρ*_0_ on *v*(*t*) for the synchronized synthetic oscillators can be evaluated by solving the constrained optimization in (17). The noise-filtering ability *ρ*_0_ of the synchronized synthetic oscillators in (17) can be obtained by decreasing *ρ* until no positive solution *V*(*e*) > 0 exists for HJI in (16) again.

If the noise-filtering ability *ρ*_0_ cannot satisfy the designer’s specification, in order to enhance the noise filtering of extrinsic noise, we need to specify the design parameters of nonlinear stochastic coupled synthetic genetic oscillators, for example, the kinetic parameters *α*_*a*_, *α*_*b*_, *α*_*c*_, *β*_*A*_, *β*_*B*_, *β*_*C*_, *γ*_*m*_, and *γ*_*p*_ in (5) to solve the constrained optimization in (17) to enhance the noise-filtering ability, i.e.,

(18) $$\rho_{0}=\min_{\alpha_{a},\alpha_{b},\alpha_{c},\beta_{A},\beta_{B},\beta_{C},\gamma_{m},\gamma_{p}}\rho$$

subject to HIJ in (16) with *V*(*e*) > 0

Before the discussion on the synchronization robustness criterion of coupled synthetic genetic oscillators, some definitions on synchronization robustness, intrinsic robustness and extrinsic robustness are given as follows:

(1) Synchronization robustness: The ability of coupled synthetic genetic oscillators to resist both intrinsic noise and extrinsic noise so that the synchronization can be maintained.

(2) Intrinsic robustness: The ability of coupled synthetic genetic oscillator to tolerate intrinsic parameter fluctuation to maintain synchronization.

(3) Extrinsic robustness: The filtering ability to attenuate the effect of environmental noise on the synchronization of coupled synthetic genetic network.

#### Remark 2

Substituting the noise-filtering ability *ρ*_0_ of (17) into (16) in Proposition 1, we get the following equivalent synchronization robustness criterion

(19) $$\begin{array}{l} \underset{\text{intrinsic robustness}}{\underset{︸}{\frac{1}{2}\left(F_{W}\left(x,s\right)+\left(C⊗I_{m}\right)G_{W}\left(x,s\right)\right)\frac{\partial^{2}V\left(e\right)}{\partial e^{2}}\left(F_{W}\left(x,s\right)+\left(C⊗I_{m}\right)G_{W}\left(x,s\right)\right)}}\\ +\underset{\text{extrinsic robustness}}{\underset{︸}{e^{T}Re+\frac{1}{4{\rho_{0}}^{2}}{\left(\frac{\partial V\left(e\right)}{\partial e}\right)}^{T}HH^{T}\left(\frac{\partial V\left(e\right)}{\partial e}\right)}}\leq \underset{\text{synchronization robustness}}{\underset{︸}{-{\left(\frac{\partial V\left(e\right)}{\partial e}\right)}^{T}\left(F\left(x,s\right)+\left(C⊗I_{m}\right)G\left(x,s\right)\right)}} \end{array}$$

The first term on the left hand side of (19) indicates the intrinsic robustness to tolerate the intrinsic parameter fluctuation in (13) because this term is induced by intrinsic noise (or random parameter fluctuation), the second and third term on the left hand side are due to the noise filtering in (14) and indicate the extrinsic robustness to filter the extrinsic noise with the noise filtering ability *ρ*_0_, and the term on the right hand side of (19) indicates the synchronization robustness of the coupled synthetic gene networks. The biological meaning of synchronization robustness criterion in (19) is that if the synchronization robustness can confer both the intrinsic robustness to tolerate intrinsic parameter fluctuation and extrinsic robustness to filter the environmental noise, then the coupled synthetic networks will synchronize with a noise filtering ability *ρ*_0_. If the synchronization robustness criterion in (19) is violated, then the synchronization of coupled synthetic gene networks may not be achieved due to the intrinsic parameter fluctuation and extrinsic noise.

In general, it is still very difficult to solve the second-order HJI in (16) with *V*(*e*) > 0 and *V*(0) = 0 to guarantee robust synchronization of nonlinear stochastic coupled synthetic genetic oscillators with a prescribed attenuation level *ρ* under intrinsic kinetic parameters fluctuations and extrinsic environmental molecular noise or to solve the constrained minimization in (18) for robust synchronization design to achieve the optimal molecular noise filtering of the synchronized coupled synthetic oscillators. Recently, the fuzzy dynamic model has been widely used to interpolate several local dynamic models to efficiently approximate a nonlinear dynamic system [32,38,39]. Hence, in this situation, we employ the T-S fuzzy model to interpolate several linear synthetic stochastic oscillators at different local operation points to efficiently and globally approximate the error dynamic in (13), so that the analysis and design procedure for robust synchronization of nonlinear stochastic coupled synthetic genetic oscillators can be simplified.

### Robust synchronization design of synthetic genetic oscillators via t-s fuzzy methodology

In this study, the T-S fuzzy method is employed to simplify the analysis and design procedure for robust synchronization of nonlinear stochastic coupled synthetic oscillators under intrinsic kinetic parameter fluctuations and extrinsic environmental molecular noise. The T-S fuzzy model for the synchronization error dynamics is described by fuzzy if-then rules. The *k*th rule of the fuzzy model for the synchronization error dynamics for cell *i* in (12) is proposed in the following form [30,32,38,39]:

**Rule*k*:** If *z*_1,*i*_(*t*) is *F*_*k*1_ and *z*_2,*i*_(*t*) is *F*_*k*2_ … and *z*_*g*,*i*_(*t*) is *F*_*kg*_, then

(20) $$\begin{array}{c} de_{i}=\left(A_{k}e_{i}+\sum_{j=1}^{N}c_{\mathrm{ij}}B_{k}e_{j}+H_{i}v_{i}\right)dt\\ \;+\left(A_{\mathrm{Wk}}e_{i}+\sum_{j=1}^{N}c_{\mathrm{ij}}B_{\mathrm{Wk}}e_{j}\right)dw_{i} \end{array}$$

for *k* = 1, 2, ⋯, *L*, where *z*_*g*,*i*_ is the element of premise variables of the *i*th coupled oscillation system, i.e., *z*_*i*_ = *z*_1,*i*_, …, *z*_*g*,*i*_^*T*^; *F*_*kg*_ is the fuzzy set; *A*_*k*_, *B*_*k*_, *A*_*Wk*_, and *B*_*Wk*_ are the fuzzy system matrices; *L* is the number of if-then rules; and *g* is the number of premise variables. The physical meaning of fuzzy rule *k* is that if the premise variables *z*_1,*i*_(*t*), *z*_2,*i*_(*t*), ⋯, *z*_*g*,*i*_(*t*) are with the fuzzy sets *F*_*k*1_, *F*_*k*2_, ⋯, *F*_*kg*_, then the synchronization error dynamics in (12) can be represented by interpolating the linearized synchronization error dynamics in (20) via the fuzzy basis. The fuzzy synchronization error dynamics in (20) is referred as follows [32,40,46]:

(21) $$\begin{array}{c} de_{i}=\sum_{k=1}^{L}\mu_{k,i}\left(z_{i}\right)\left(\left(A_{k}e_{i}+\sum_{j=1}^{N}c_{\mathrm{ij}}B_{k}e_{j}+H_{i}v_{i}\right)dt\right)\\ \;+\left(A_{\mathrm{Wk}}e_{i}+\sum_{j=1}^{N}c_{\mathrm{ij}}B_{\mathrm{Wk}}e_{j}\right)dw_{i} \end{array}$$

where $\mu_{k,i}\left(z_{i}\right)≜\frac{\prod_{j=1}^{g}F_{\mathrm{kj}}\left(z_{j,i}\right)}{\sum_{k=1}^{L}\prod_{j=1}^{g}F_{\mathrm{kj}}\left(z_{j,i}\right)}$, *F*_*kj*_(*z*_*j*,*i*_) is the grade of membership of *z*_*j*,*i*_(*t*) in *F*_*kj*_ or the possibility function of *z*_*j*,*i*_(*t*) in *F*_*kj*_, and *μ*_*k*_(*z*_*i*_) is called fuzzy basis function for *k* = 1, 2, …, *L*. The denominator or $\sum_{k=1}^{L}\prod_{j=1}^{g}F_{\mathrm{kj}}\left(z_{j,i}\right)$ in the above fuzzy basis function is only for normalization, so that the total sum of fuzzy basis is $\sum_{k=1}^{L}\mu_{k,i}\left(z_{i}\right)=1$. The physical meaning of (21) is that the fuzzy stochastic system interpolates *L* local linear stochastic systems through nonlinear basis *μ*_*k*_(*z*_*i*_) to approximate the nonlinear stochastic system in (13). In this situation, the nonlinear stochastic coupled oscillation systems in (13) can be represented by the fuzzy interpolation system as follows:

(22) $$\begin{array}{c} de=\left(F\left(x,s\right)+\left(C⊗I_{m}\right)G\left(x,s\right)+Hv\right)dt+\left(F_{W}\left(x,s\right)+\left(C⊗I_{m}\right)G_{W}\left(x,s\right)\right)dw\\ \;=\sum_{k=1}^{L}\mu_{k}\left(z\right)\left(\left(\left(I_{N}⊗A_{k}+C⊗B_{k}\right)e+Hv\right)dt+\left(\left(I_{N}⊗A_{\mathrm{Wk}}+C⊗B_{\mathrm{Wk}}\right)e\right)dw\right) \end{array}$$

where *μ*_*k*_(*z*) = *diag*(*μ*_*k*,1_(*z*_1_), …, *μ*_*k*,*N*_(*z*_*N*_)) and *z* = *z*_1_, …, *z*_*N*_^*T*^.

#### Remark 3

In [39], Takagi and Sugeno have proposed the systematic method to build T-S fuzzy model for nonlinear function approximation by the system identification tool, i.e. the local system matrix *A*_*k*_, *B*_*k*_, *A*_*Wk*_, and *B*_*Wk*_ in (21) or (22) can be identified by least square estimation method. On the other hand, many studies have proved that the T-S fuzzy model can approximate a continuous function with any degree of accuracy. Actually, there is still some fuzzy approximation error in (22). In the robust synchronization control design, for simplicity, the fuzzy approximation error can be merged into the external noise, which could be efficiently attenuated by the proposed *H*_∞_ robust synchronization control design in the sequel.

After investigating the approximation of nonlinear stochastic coupled synthetic oscillators by the fuzzy interpolation method, in order to avoid solving the nonlinear constrained optimization problem in (18) for the robust synchronization design problem of coupled synthetic oscillators under intrinsic kinetic parameter fluctuation and extrinsic environmental molecular noise, the measurement procedure for the noise-filtering ability of synchronized synthetic genetic oscillators could also be simplified by the fuzzy approximation method. Then, we get the following result.

#### Proposition 2

If there exists a positive definite symmetric matrix *P*>0 solving the following LMIs,

(23) $$\left[\begin{array}{cc} \begin{array}{l} R+P(I_{N}⊗A_{k}+C⊗B_{k})+{\left(I_{N}⊗A_{k}+C⊗B_{k}\right)}^{T}P\\ +{\left(I_{N}⊗A_{\mathrm{Wk}}+C⊗B_{\mathrm{Wk}}\right)}^{T}P(I_{N}⊗A_{\mathrm{Wk}}+C⊗B_{\mathrm{Wk}}) \end{array} & PH\\ H^{T}P & -\rho^{2}I \end{array}\right]<0$$

for *k* = 1, 2, …, *L*, then the noise-filtering level *ρ* in (14) holds, or the intrinsic kinetic parameter fluctuations are tolerated by the synchronized synthetic genetic oscillators, and the influence of extrinsic environmental molecular noise *v*(*t*) on the synchronized synthetic oscillation systems in (13) is less than or equal to a prescribed filtering level *ρ*.

#### Proof: See Additional file 1: Appendix B

Therefore, the optimal noise-filtering design of synchronized oscillation systems obtained by solving the HJI-constrained optimization problem in (18) could be replaced by solving the following constrained optimizations, respectively

(24) $$\rho_{0}=\min_{\alpha_{a},\alpha_{b},\alpha_{c},\beta_{A},\beta_{B},\beta_{C},\gamma_{m},\gamma_{p}}\rho$$

subject to *P*>0 and LMIs in (23)

#### Remark 4

(i)  If the prescribed noise-filtering level *ρ* is prescribed by a biological engineer, a robust synchronization design would involve specifying the design parameters *α*_*a*_, *α*_*b*_, *α*_*c*_, *β*_*A*_, *β*_*B*_, *β*_*C*_, *γ*_m_, and *γ*_*p*_ of the synthetic gene oscillators in *A*_*k*_, so that the LMIs in (23) have a positive solution *P*>0 in Proposition 2. If we want to achieve optimal filtering of extrinsic noise for the synchronized synthetic oscillators, some design parameters need to be specified for coupled synthetic oscillators to achieve the constrained optimization in (24).

(ii)  In this study, the fuzzy approximation method in (21) or (22) is only employed to simplify the analysis and design procedure via solving *P*>0 for LMIs in (23) instead of solving *V*(*e*) > 0 for HJI in (16) directly. Further, based on the fuzzy interpolation of local linear systems, i.e., replacing *F*(*x*, *s*), *G*(*x*, *s*), *F*_*W*_(*x*, *s*) and *G*_*W*_(*x*, *s*) by the fuzzy approximations in (22), in Proposition 2, *V*(*e*) = *e*^*T*^*Pe* is employed to solve the HJI (16) in Proposition 1. The HJI in Proposition 1 is replaced with a set of LMIs in Proposition 2 and we only need to solve *P*>0 for LMIs to guarantee the coupled synthetic genetic oscillators have a noise filtering level *ρ*.

(iii)  In general, the constrained optimization problems in (24) are called eigenvalue problem [41], which can be efficiently solved by the MATLAB LMI toolbox.

(iv)  In addition to the robust oscillation synchronization, the proposed method can be applied to robust synchronization design of coupled synthetic gene networks with any kind of dynamic behavior.

(v)  In the fuzzy approximation case, the synchronization robustness criterion in (19) is equivalent to the following

(25) $$\begin{array}{l} \underset{\text{local intrinsic robustness}}{\underset{︸}{{\left(I_{N}⊗A_{\mathrm{Wk}}+C⊗B_{\mathrm{Wk}}\right)}^{T}P\left(I_{N}⊗A_{\mathrm{Wk}}+C⊗B_{\mathrm{Wk}}\right)}}+\underset{\text{local extrinsic robustness}}{\underset{︸}{R+\frac{1}{\rho_{0}^{2}}PHH^{T}P}}\\ \leq -\underset{\text{local synchronization robustness}}{\underset{︸}{\left(P\left(I_{N}⊗A_{k}+C⊗B_{k}\right)+{\left(I_{N}⊗A_{k}+C⊗B_{k}\right)}^{T}P\right)}} \end{array}$$

for *k* = 1, 2, …, *L* which is equivalent to (23) with *ρ* being replace by *ρ*_0_. The biological meaning of synchronization robustness criterion in (25) is that if the local synchronization robustness of local coupled synthetic genetic oscillators can confer local intrinsic robustness to tolerate local intrinsic parameter fluctuation and local extrinsic robustness to filter external noise, then the coupled synthetic genetic oscillators can be synchronized with a noise filtering ability *ρ*_0_. If the synchronization robustness criterion in (25) is violated, then the synchronization of coupled synthetic genetic oscillators may not be achieved due to intrinsic parameter fluctuation and extrinsic noise. In general, if the design parameters of coupled synthetic genetic oscillators are specified so that the eigenvalues of local coupled system matrix *I*_*N*_ ⊗ *A*_*Wk*_ + *C* ⊗ *B*_*Wk*_ are far in the left hand side of complex s-domain (i.e. with more negative real part), then the coupled synthetic genetic networks are more easy to synchronize in spite of intrinsic parameter fluctuation and extrinsic noise.

### Robust synchronization of synthetic genetic oscillators by external control input

If robust synchronizations of coupled synthetic genetic oscillators cannot be achieved spontaneously via the parameter design in the above sections, then a control strategy is needed from external stimulation inputs to improve the robust synchronization of coupled synthetic genetic oscillators. External stimulation inputs are known to play an important role in the synchronization of biological rhythms. Recently, several methods of periodic stimulation for synchronization of nonlinear oscillators have been introduced [7-9,17]. However, even simple methods may show enormous complexity in the control scheme for synchronization of nonlinear stochastic coupled oscillators. In this study, based on nonlinear *H*_∞_ stochastic control theory, an input control strategy is introduced to enhance the robust synchronization. If AI is injected into a common medium to increase the average concentration of AI protein in the exteracellular environment, which in turn increases the cellular communication of coupled oscillation systems, then the dynamics of the signaling molecule AI in the cellular environment, as shown in (3), should be modified as

(26) $$\begin{array}{c} \frac{dx_{\mathrm{Si}}\left(t\right)}{dt}=-d_{\mathrm{se}}x_{\mathrm{Si}}\left(t\right)+\beta_{s}x_{\mathrm{Ai}}\left(t\right)\\ \;-\eta_{s}\left(Q_{e}+u_{e}\right)\left(x_{\mathrm{Si}}\left(t\right)-N^{-1}\sum_{j=1}^{N}x_{\mathrm{Sj}}\left(t\right)\right) \end{array}$$

where *u*_*e*_ = *Q* represents an extracellular control input, which can be implemented via the injection of inducer AI.

For the simplicity of control design, suppose that the following control input *u*_*e*_ = *Q* is employed to improve the robust synchronization of the nonlinear stochastic coupled synthetic oscillation systems. In this situation, the synchronization error dynamics in (13) should be modified as follows:

(27) $$\begin{array}{c} de=\left(F\left(x,s\right)+\left(C_{e}\left(Q\right)⊗I_{m}\right)G\left(x,s\right)+Hv\right)dt\\ \;+\left(F_{W}\left(x,s\right)+\left(C_{e}\left(Q\right)⊗I_{m}\right)G_{W}\left(x,s\right)\right)dw \end{array}$$

where *C*_*e*_(*Q*) = (*c*_*eij*_(*Q*))_*N* × *N*_ ∈ *R*^*N* × *N*^ is the coupling configuration matrix, in which *c*_*eii*_(*Q*) = − *η*_*s*_(1 − *N*^− 1^)(*Q*_*e*_ + *Q*) if *i=j*, otherwise *c*_*eij*_(*Q*) = *η*_*s*_*N*^− 1^(*Q*_*e*_ + *Q*). Then, we can also obtain the robust synchronization control design of coupled oscillation systems under intrinsic kinetic parameter fluctuations and extrinsic environmental molecular noises as follows.

#### Corollary 3

For the nonlinear stochastic coupled synthetic genetic oscillators with an extracellular control input *u*_*e*_*=Q* in the terms of *C*_*e*_(*Q*) in (27), if there exists a positive solution *V*(*e*) > 0 with *V*(0) = 0 to the following HJI

(28) $$\begin{array}{l} e^{T}Re+{\left(\frac{\partial V\left(e\right)}{\partial e}\right)}^{T}\left(F\left(x,s\right)+\left(C_{e}\left(Q\right)⊗I_{m}\right)G\left(x,s\right)\right)+\frac{1}{4\rho^{2}}{\left(\frac{\partial V\left(e\right)}{\partial e}\right)}^{T}HH^{T}\left(\frac{\partial V\left(e\right)}{\partial e}\right)\\ +\frac{1}{2}\left(F_{W}\left(x,s\right)+\left(C_{e}\left(Q\right)⊗I_{m}\right)G_{W}\left(x,s\right)\right)\frac{\partial^{2}V\left(e\right)}{\partial e^{2}}\left(F_{W}\left(x,s\right)+\left(C_{e}\left(Q\right)⊗I_{m}\right)G_{W}\left(x,s\right)\right)<0 \end{array}$$

for a prescribed filtering level *ρ*, then the stochastic intrinsic kinetic noise can be robustly tolerated, and the influence of extrinsic environmental molecular noise *v*(*t*) on the synchronization of the nonlinear stochastic coupled synthetic oscillation systems in (27) is less than or equal to *ρ*, i.e., the inequality in (14) or (15) holds.

#### Proof: similar to the proof of proposition 1

The inequality (28) is equivalent to synchronization robustness criterion

(29) $$\begin{array}{l} \underset{\text{intrinsic robustness}}{\underset{︸}{\frac{1}{2}\left(F_{W}\left(x,s\right)+\left(C_{e}\left(Q\right)⊗I_{m}\right)G_{W}\left(x,s\right)\right)\frac{\partial^{2}V\left(e\right)}{\partial e^{2}}\left(F_{W}\left(x,s\right)+\left(C_{e}\left(Q\right)⊗I_{m}\right)G_{W}\left(x,s\right)\right)}}\\ +\underset{\text{extrinsic robustness}}{\underset{︸}{e^{T}Re+\frac{1}{4\rho^{2}}{\left(\frac{\partial V\left(e\right)}{\partial e}\right)}^{T}HH^{T}\left(\frac{\partial V\left(e\right)}{\partial e}\right)}}\leq \underset{\text{synchronization robustness}}{\underset{︸}{-{\left(\frac{\partial V\left(e\right)}{\partial e}\right)}^{T}\left(F\left(x,s\right)+\left(C_{e}\left(Q\right)⊗I_{m}\right)G\left(x,s\right)\right)}} \end{array}$$

The physical meaning of synchronization robustness criterion in (29) is that if we can specify control parameter *Q* to improve the synchronization robustness to provide more intrinsic robustness and more extrinsic robustness to tolerate more intrinsic parameter fluctuation and filter more extrinsic noise, then the robust synchronization of the nonlinear stochastic coupled synthetic genetic oscillators in (27) can be guaranteed. Similarly, the optimal noise-filtering design of synchronized oscillation systems by the extracellular control input in (27) can be achieved by solving the following constrained optimization problem:

(30) $$\rho_{0}=\min_{Q}\rho$$

subject to *V*(*e*) > 0 and HJI in (28)

In general, it is still very difficult to specify the control parameter *u*_*e*_*=Q* to solve the HJI-constrained optimization in (30) for achieving the optimal noise filtering for synchronized synthetic genetic oscillators. Therefore, the fuzzy approximation method is again employed to simplify the control design procedure. Based on the fuzzy approximation method, the following fuzzy interpolation system is employed to approach the nonlinear stochastic coupled oscillation systems in (27):

(31) $$\begin{array}{c} de=\sum_{k=1}^{L}\mu_{k}\left(z\right)\left(\left(\left(I_{N}⊗A_{k}+C_{e}\left(Q\right)⊗B_{k}\right)e+Hv\right)dt\right)\\ \;+\left(\left(I_{N}⊗A_{\mathrm{Wk}}+C_{e}\left(Q\right)⊗B_{\mathrm{Wk}}\right)e\right)dw \end{array}$$

Applying the fuzzy approximation method, the external signal control design can be obtained as described in the following corollary, for robust filtering of synchronized oscillation systems with intrinsic kinetic parameter fluctuations and extrinsic environmental molecular noise.

#### Corollary 4

For stochastic synchronized oscillation systems, if there exists a symmetric solution *P* > 0 to the following LMIs for a prescribed noise-filtering level *ρ*

(32) $$\left[\begin{array}{cc} \begin{array}{l} R+P(I_{N}⊗A_{k}+C_{e}(Q)⊗B_{k})+{\left(I_{N}⊗A_{k}+C_{e}\left(Q\right)⊗B_{k}\right)}^{T}P\\ +{\left(I_{N}⊗A_{\mathrm{Wk}}+C_{e}\left(Q\right)⊗B_{\mathrm{Wk}}\right)}^{T}P(I_{N}⊗A_{\mathrm{Wk}}+C_{e}(Q)⊗B_{\mathrm{Wk}}) \end{array} & PH\\ H^{T}P & -\rho^{2}I \end{array}\right]<0$$

for *k* = 1, 2, ⋯, *L*, then intrinsic parametric noise can be tolerated and the effect of extrinsic molecular noise *v*(*t*) on the synchronization of nonlinear stochastic coupled oscillation systems is less than or equal to a prescribed filtering level *ρ*.

#### Proof: similar to the proof of proposition 2

The physical meaning of Corollary 4 is that if we can select a control parameter *Q*, such that the LMIs in (32) have a positive definite solution *P* > 0, then the robust synchronization with a prescribed noise filtering level *ρ* on extrinsic environmental molecular noise is guaranteed for the nonlinear stochastic coupled synthetic genetic oscillators. If we specify control parameter *Q* so that the eigenvalues of local system matrix of *I*_*N*_ ⊗ *A*_*k*_ + *C*_*e*_(*Q*) ⊗ *B*_*k*_ of coupled synthetic gene oscillators have more negative real part (i.e. in far left hand complex s-domain), the coupled synthetic gene oscillators are with more robust synchronization to tolerate more intrinsic parameter fluctuations and to filter more extrinsic noise.

Similarly, based on the fuzzy approximation method, an optimal noise-filtering design of synchronized oscillation systems by using the extracellular control input in (30) can be achieved by solving the following constrained optimization problem:

(33) $$\rho_{0}=\min_{Q}\rho$$

subject to *V*(*e*) > 0 and LMIs in (32)

The physical meaning of the constrained optimization in (33) is that if we can select a control parameter *Q* through the inducer concentration control method to solve the constrained optimization problem, we can achieve both robust synchronization against intrinsic kinetic parameter fluctuations and optimal filtering against external environmental molecular noise on the synchronization by using the external control signal in the coupled synthetic oscillation systems.

The design procedure of external inducer control for robust synchronization of the coupled network is summarized as follows.

(1) Consider a synthetic genetic network of *N* coupled oscillators with intrinsic kinetic parameter fluctuations and extrinsic environmental molecular noise.

(2) Given the prescribed disturbance attenuation level *ρ*.

(3) Represent the nonlinear stochastic synchronization error dynamic by the T-S fuzzy synchronization error dynamic model, using the interpolation of several local linear stochastic systems.

(4) Specify *Q* to solve LMI in (32) with the help of LMI toolbox in MATLAB so that *N* coupled synthetic genetic oscillators can be synchronized with a prescribed noise filtering level *ρ*.

### Design examples *in silico* for robust synchronization design in the genetic oscillation systems

In this section, we provide a simulated example to illustrate the design procedure of robust synchronization of the nonlinear stochastic coupled synthetic oscillation systems and to confirm the performance of the robust synchronization of proposed method against intrinsic kinetic parameter fluctuations and extrinsic environmental molecular noise.

**Figure 2:**
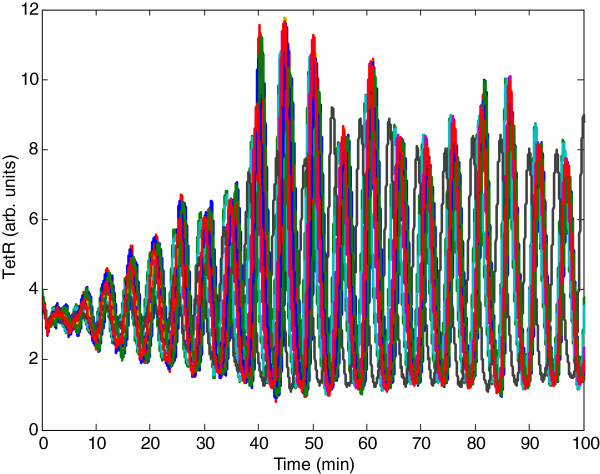
**Ten coupled genetic oscillators.** The parameter values in (1), (2), and (3) are set as follows: *α*_*a*_ = *α*_*b*_ = *α*_*c*_ = 216, *α*_*S*_ = 20, *μ* = 1.2, *μ*_*S*_ = 1, *n* = 2, *γ*_*S*_ = 1, *η*_*S*_ = 2, *β*_*S*_ = 0.1, *β*_*A*_ = *β*_*B*_ = *β*_*C*_ = 1, *γ*_*m*_ = 6.9315, *γ*_*p*_ = 1.1552 and *Q*_*e*_ = 0.09 [1]. Suppose the nonlinear stochastic coupled synthetic oscillators suffer from stochastic parameter fluctuations as shown in (8) with *Δα*_*a*_ = *Δα*_*b*_ = *Δα*_*c*_ = 2.16, *Δα*_*S*_ = 0.2, *Δβ*_*A*_ = *Δβ*_*B*_ = *Δβ*_*C*_ = 0.01, *Δβ*_*S*_ = 0.001, *Δη*_*S*_ = 0.02, *Δγ*_*m*_ = 0.06, *Δγ*_*p*_ = 0.01, and *Δγ*_*S*_ = 0.01. For the convenience of simulation, we assume that the extrinsic molecular noise *v*_1_~*v*_10_ is independent Gaussian white noise with a mean of zero and standard deviation of 0.02. It can be seen that coupled synthetic oscillators cannot achieve synchronization under these intrinsic kinetic parameter fluctuations and extrinsic molecular noise.

**Figure 3:**
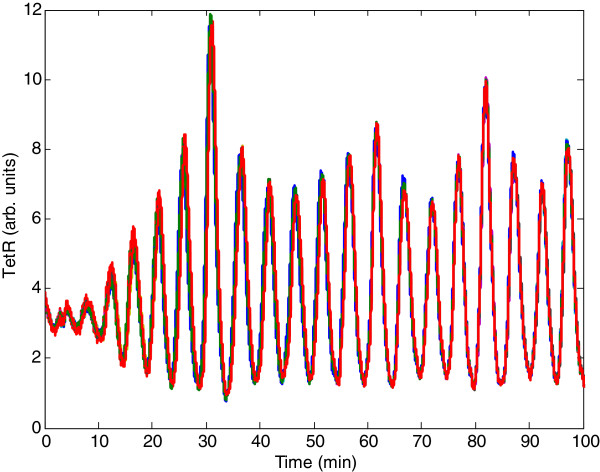
**The robust synchronization result of ten coupled synthetic oscillators in Figure**2**, by external control with *Q*** **=** **0.66.** Based on a Monte Carlo simulation with 100 runs, the noise filtering level is given by (*E* ∫ _0_^100^*e*^*T*^(*t*)*Re*(*t*)*dt*)/(*E* ∫ _0_^100^*v*^*T*^(*t*)*v*(*t*)*dt*) ≈ 0.19^2^ < 0.56^2^.

The purpose of this example is to demonstrate the effectiveness of the theoretical synchronization result of synthetic gene oscillators in mimicking real biological oscillator systems. We consider a synthetic genetic network of *N*=10 coupled synthetic genetic oscillators with intrinsic kinetic parameter fluctuations and extrinsic environmental molecular noise in (5). The simulation results are shown in Figure 2. It can be seen that the parameter set of the coupled synthetic oscillator network, as listed below the figure, cannot make the whole network synchronize spontaneously. Suppose we want to specify a control parameter *Q* (which is proportional to the density of inducer AI) in (26) to compensate for the inefficiency of coupling between the synthetic genetic oscillators from the quasi-steady-state point of view. The design procedure first begins with representing the nonlinear stochastic synchronization error dynamic in (22) by the T-S fuzzy synchronization error dynamic model in (31), using the interpolation of several linear stochastic systems as presented in Additional file 1: Appendix C. According to the fuzzy approximation and Corollary 4, our control design problem is how to specify *Q* (i.e. the corresponding density of inducer AI), so that the ten coupled synthetic genetic oscillators have a positive solution *P* > 0 with a prescribed noise filtering level *ρ* = 0.56 to guarantee robust synchronization under intrinsic kinetic parameter fluctuations and extrinsic environmental molecular noise. The LMI toolbox in MATLAB can then be used to significantly simplify the system analysis and design procedure. With *Q* = 0.66 solved from LMIs in (32), the outputs of the coupled gene network of ten synthetic oscillators under intrinsic parametric fluctuations and extrinsic noise are shown in Figure 3. It can be seen that the coupled synthetic genetic oscillators have robust synchronizability to achieve the synchronous behavior despite the effect of uncertain initial state, intrinsic kinetic parameter fluctuations, and extrinsic environmental molecular noise on the host cell. According to a Monte Carlo simulation with 100 runs, the noise-filtering level of the coupled gene network is given by (*E* ∫ _0_^100^*e*^*T*^(*t*)*Re*(*t*)*dt*)/(*E* ∫ _0_^100^*v*^*T*^(*t*)*v*(*t*)*dt*) ≈ 0.19^2^ < 0.56^2^. It can be clearly seen that based on our proposed design method, the coupled gene network cannot only tolerate kinetic parameter variations but also attenuate the extrinsic molecular noise below a desired level to achieve a robust synchronization.

## Discussion

The cell is the functional unit of all living things, either unicellular or multicellular. A cell can sense many different signals from the internal or external context and can respond to the constantly changing environment via appropriate cellular processes. Also, cells can interact with each other via cell-to-cell communication and achieve specific physiological functions essential for life in a cooperative manner. However, many fundamental questions remain regarding how cellular phenomena arise from the interactions between genes and proteins, what features make the cell operate reliably in diverse conditions, and how the cell is responsible for these operations. To gain insight into these questions, one can construct the underlying mechanisms that constitute the web of interactions. This idea is useful to separate a complicated network into many simpler ones, which can work independently but also cooperate with each other. It may not only enhance our understanding of collective behavior particularly via synchronization but may also establish a foundation to design robust implementation of coupled synthetic gene networks [47,48].

In this paper, we consider a nonlinear stochastic coupled network with two or more coupled synthetic oscillators. By transforming nonlinear stochastic coupled network dynamics into synchronization error dynamics, we can use Lyapunov’s direct method to infer a sufficient condition required for robustness of the nonlinear synchronized network. Assuming that each synthetic oscillator suffers from intrinsic kinetic parameter fluctuations and extrinsic molecular noise, robust synchronization performance is defined as the effect of extrinsic molecular noise upon the synchronization error. Based on this definition, robust synchronization performance of a nonlinear coupled network can be calculated by solving an associated HJI-constrained optimization problem. We also show that nonlinear coupled networks with robust synchronization performance are also *synchronizable*. Based on this synchronization performance, we propose a procedure for designing or compensating a coupled network with two or more coupled synthetic oscillator through a given connected topology toward a desired robust synchronization performance. Using the proposed method, the coupled synthetic oscillators cannot only tolerate kinetic parameter variations but also attenuate the extrinsic molecular noise below a desired level to achieve a desired robust synchronization. However, the HJI-constrained optimization problem is difficult to solve directly by any analytical or numerical method because of the complexity of nonlinear synchronization error dynamics. Hence, we employ a T–S fuzzy model to solve the HJI easily and indirectly. The T–S fuzzy model has been widely applied to approximate nonlinear systems by interpolating several local linearized systems. Here, we use the T–S fuzzy model to approximate the nonlinear stochastic synchronization error dynamics. By using the T–S fuzzy model and choosing the appropriate Lyapunov function, the HJI-constrained optimization for calculating the robust synchronization performance of a nonlinear coupled network is reduced to an equivalent LMI-constrained optimization problem, which can be solved efficiently by MATLAB’s LMI toolbox.

Recently a simple synthetic device was engineered in a cell, and several cells were then combined, so that their connections allowed the construction of a more complex synthetic gene circuit, i.e., so-called multi-cellular engineered networks. This approach not only uses cellular consortia as an efficient way of engineering complex gene circuits, but also demonstrates the great potential for reutilization of small parts of the gene circuit. In such situations, our proposed evaluation framework may offer a possible guideline for the design or compensation of such coupled networks with a given connected topology toward a desired collective behavior.

## Conclusions

In this study, several robust synchronization criteria and designs are proposed for a population of synthetic genetic oscillators in order to exploit an emergent synchronization phenomenon by quorum sensing molecules under intrinsic kinetic parameter fluctuations and extrinsic molecular noise. When the synchronization of nonlinear stochastic coupled synthetic genetic oscillators cannot be maintained, a robust *H*_∞_ control scheme is developed to enhance synchronization by adding external control to increase the cell-to-cell communication through quorum sensing. This study enhances our understanding in this area in the following ways: (a) nonlinear stochastic systems are employed to model the coupled synthetic genetic oscillators with intrinsic kinetic parameter fluctuations, extrinsic molecular noises on the host cells and quorum sensing molecules; (b) two robust synchronization criteria (19) and (25) of coupled synthetic oscillators are developed from the nonlinear stochastic filtering point of view, so that we can gain more insight into the robust synchronization mechanism from a systematic perspective. If these robust synchronization criterion cannot be guaranteed, robust synchronization control schemes via selecting adequate kinetic parameters and inducer concentration are also developed to improve the synchronization robustness of coupled synthetic genetic oscillators; and (c) the fuzzy approximation is employed to approximate the nonlinear stochastic synchronization error model by interpolating several linear stochastic systems, so that the powerful LMI toolbox in MATLAB can be used to significantly simplify the system analysis and design procedure for robust synchronization of coupled synthetic gene networks, which is very important for the emergent phenomenon of synthetic gene networks through quorum sensing at the molecular level. In addition, the proposed robust synchronization design and control scheme for nonlinear stochastic coupled genetic oscillators can be easily applied to the robust synchronization problem of other coupled genetic networks as a cellular consortium.

## Competing interests

The authors declare that they have no competing interests.

## Authors’ contributions

BSC formulated the research topic and carried out some robust synchronization control designs. CYH participated in some control designs and performed simulation. Both authors read and approved the final manuscript.

## Supplementary Material

Additional file 1 Supplementary Appendix This file contains appendices A, B and C. Click here for file
