# Supplementary material for: Robust synchronization control scheme of a population of nonlinear stochastic synthetic genetic oscillators under intrinsic and extrinsic molecular noise via quorum sensing
Source: BMC Syst Biol. 2012 Oct 26;6:136. doi: 10.1186/1752-0509-6-136 (PMC3554485; doi:10.1186/1752-0509-6-136)
Supplement: Additional file 1 Supplementary Appendix — This file contains appendices A, B and C. [file 1752-0509-6-136-S1.pdf]

Appendix

Appendix A: *Proof of Proposition 1*

Consider the following equality

$$E\int_0^{t_f} e^T Redt = EV\left(e(0)\right) - EV\left(e(t_f)\right) + E\int_0^{t_f}\left(e^T Re + \frac{dV(e)}{dt}\right)dt \qquad \text{(A1)}$$

By Ito formula (Zhang *et al.*, 2005), we get

$$\begin{aligned} &dV(e)\\ &=\left(\frac{\partial V(e)}{\partial e}\right)^T \left(\left(F(x,s)+\left(C\otimes I_m\right)G(x,s)+Hv\right)dt+\left(F_W(x,s)+\left(C\otimes I_m\right)G_W(x,s)\right)dw\right)\\ &\quad+\frac{1}{2}\left(F_W(x,s)+\left(C\otimes I_m\right)G_W(x,s)\right)^T\frac{\partial^2 V(e)}{\partial e^2}\left(F_W(x,s)+\left(C\otimes I_m\right)G_W(x,s)\right)dt \end{aligned} \qquad \text{(A2)}$$

Substituting (A2) into (A1) and by the fact $EdW = 0$, $EV\left(e(t_f)\right)\geq 0$, we get

$$\begin{aligned} E\int_0^{t_f} e^T Redt \leq EV\left(e(0)\right)+E\int_0^{t_f}\Bigg(& e^T Re+\left(\frac{\partial V(e)}{\partial e}\right)^T\left(F(x,s)+\left(C\otimes I_m\right)G(x,s)+Hv\right)\\ &+\frac{1}{2}\left(F_W(x,s)+\left(C\otimes I_m\right)G_W(x,s)\right)^T\frac{\partial^2 V(e)}{\partial e^2}\left(F_W(x,s)+\left(C\otimes I_m\right)G_W(x,s)\right)\Bigg)dt \end{aligned} \qquad \text{(A3)}$$

By the fact that

$$\mathbf{a}^T\mathbf{b} = 2\left(\frac{1}{2\rho}\mathbf{a}\right)^T\left(\rho\mathbf{b}\right)\leq\frac{1}{4\rho^2}\mathbf{a}^T\mathbf{a}+\rho^2\mathbf{b}^T\mathbf{b} \qquad \text{(A4)}$$

for any $\rho > 0$ and vectors $\mathbf{a}$ and $\mathbf{b}$. Then, we get the following inequality

$$\begin{aligned} &E\int_0^{t_f} e^T Redt \leq EV\left(e(0)\right)\\ &+E\int_0^{t_f}\Bigg(e^T Re+\left(\frac{\partial V(e)}{\partial e}\right)^T\left(F(x,s)+\left(C\otimes I_m\right)G(x,s)\right)+\frac{1}{4\rho^2}\left(\frac{\partial V(e)}{\partial e}\right)^T HH^T\left(\frac{\partial V(e)}{\partial e}\right)\\ &\qquad+\frac{1}{2}\left(F_W(x,s)+\left(C\otimes I_m\right)G_W(x,s)\right)\frac{\partial^2 V(e)}{\partial e^2}\left(F_W(x,s)+\left(C\otimes I_m\right)G_W(x,s)\right)\Bigg)dt \end{aligned}$$

where $\mathbf{a} = H^T\dfrac{\partial V(e)}{\partial e}$ and $\mathbf{b} = v$

By the inequality in (19), we get

$$E\int_0^{t_f} e^T R e dt \le EV\left(e(0)\right) + E\rho^2 \int_0^{t_f} v^T v dt \tag{A5}$$

If coupled synthetic genetic network is free of extrinsic noise, i.e., $v(t)=0$, then from (A5), we get

$$E\int_0^{t_f} e^T R e dt \le EV\left(e(0)\right) \tag{A6}$$

Since $EV(e(0))$ is a constant, from (A6), we get $E(e(t)) \to 0$ as $t_f \to \infty$ and intrinsic parameter fluctuation is tolerated.

Appendix B: *Proof of Proposition 2*

First, we use the following fuzzy interpolation system

$$de = \sum_{k=1}^{N} \mu_k(z)\Big(\big((I_N \otimes A_k + C \otimes B_k)e + Hv\big)dt + \big((I_N \otimes A_{Wk} + C \otimes B_{Wk})e\big)dw\Big)$$

in (24) to replace (16). Following the proof of Proposition 1 in Appendix A, we get the following result

$$\begin{aligned} E\int_0^{t_f} e^T Redt &\le EV\big(e(0)\big) - EV\big(e(t_f)\big) \\ &+ E\int_0^{t_f}\left(e^T Re + \sum_{k=1}^{L}\mu_k(z)\left(\left(\frac{\partial V(e)}{\partial e}\right)^T \big((I_N \otimes A_k + C \otimes B_k)e + Hv\big)\right.\right. \\ &\left.\left. + \frac{1}{2}e^T\left(I_N \otimes A_{Wk} + C \otimes B_{Wk}\right)\frac{\partial^2 V(e)}{\partial e^2}\left(I_N \otimes A_{Wk} + C \otimes B_{Wk}\right)e\right)dt\right) \end{aligned} \quad \text{(B1)}$$

By the facts that $V\big(e(t_f)\big) \ge 0$, and

$$\left(\frac{\partial V(e)}{\partial e}\right)^T Hv \le \frac{1}{4\rho^2}\left(\frac{\partial V(e)}{\partial e}\right)^T HH^T\left(\frac{\partial V(e)}{\partial e}\right) + \rho^2 v^T v$$

we get the following result from (B1)

$$\begin{aligned} E\int_0^{t_f} e^T Redt &\le EV\big(e(0)\big) \\ &+ E\int_0^{t_f}\left(e^T Re + \sum_{k=1}^{L}\mu_k(z)\left(\left(\frac{\partial V(e)}{\partial e}\right)^T (I_N \otimes A_k + C \otimes B_k)e + \frac{1}{4\rho^2}\left(\frac{\partial V(e)}{\partial e}\right)^T HH^T\left(\frac{\partial V(e)}{\partial e}\right)\right.\right. \\ &\left.\left. + \rho^2 v^T v + \frac{1}{2}e^T\left(I_N \otimes A_{Wk} + C \otimes B_{Wk}\right)^T\frac{\partial^2 V(e)}{\partial e^2}\left(I_N \otimes A_{Wk} + C \otimes B_{Wk}\right)e\right)dt\right) \end{aligned} \quad \text{(B2)}$$

If we choose $V(e) = e^T Pe$, then $\frac{\partial V(e)}{\partial e} = 2Pe$, $\frac{\partial^2 V(e)}{\partial e^2} = 2P$, then we get the following result from (B2)

$$\begin{aligned} E\int_0^{t_f} e^T Redt &\le Ee(0)^T Pe(0) \\ &+ E\sum_{k=1}^{L}\mu_k(z)\int_0^{t_f} e^T\Big(R + P\left(I_N \otimes A_k + C \otimes B_k\right) + \left(I_N \otimes A_k + C \otimes B_k\right)^T P \\ &+ \left(I_N \otimes A_{Wk} + C \otimes B_{Wk}\right)^T P\left(I_N \otimes A_{Wk} + C \otimes B_{Wk}\right) + \frac{1}{\rho^2}PHH^T P\Big)edt + \rho^2 v^T vdt \end{aligned} \quad \text{(B3)}$$

If

$$R + P\left(I_N \otimes A_k + C \otimes B_k\right) + \left(I_N \otimes A_k + C \otimes B_k\right)^T P$$
$$+ \left(I_N \otimes A_{Wk} + C \otimes B_{Wk}\right)^T P\left(I_N \otimes A_{Wk} + C \otimes B_{Wk}\right) + \frac{1}{\rho^2} PHH^T P < 0 \quad \text{(B4)}$$

then we get

$$E\int_0^{t_f} e^T R e dt \le E e(0)^T P e(0) + E\rho^2 \int_0^{t_f} v^T v dt$$

which is (18) and will be reduced to (17) if $e(0) = 0$.

Therefore, if the LMIs in (B4) hold, then the noise filtering ability ρ on the synchronization of the coupled oscillation systems is achieved. By Schur complement, the inequalities in (B4) are equivalent to the LMIs in (25), i.e. if the LMIs in (25) have a common solution $P > 0$, then the synchronization of nonlinear stochastic coupled synthetic oscillation systems in (8) has a filtering level ρ against the extrinsic noises.

Appendix C:

The fuzzy approximation is employed to approximate the nonlinear stochastic synchronization error dynamic by interpolating several local linear stochastic systems as follows:

$$\begin{aligned} de &= \left(F(x,s)+\left(C\otimes I_m\right)G(x,s)+Hv\right)dt+\left(F_W(x,s)+\left(C\otimes I_m\right)G_W(x,s)\right)dw \\ &= \sum_{k=1}^{16}\mu_k(z)\Big(\big(\left(\left(I_N\otimes A_k+C\otimes B\right)e+Hv\right)dt+\left(\left(I_N\otimes A_{Wk}+C\otimes B_W\right)e\right)dw\Big) \end{aligned}$$

where

$$A_1=\begin{bmatrix} -6.93 & 0 & 0 & 0 & 0 & -86.4 & 0 \\ 0 & -6.93 & 0 & -86.4 & 0 & 0 & 0 \\ 0 & 0 & -6.93 & 0 & -0.15 & 0 & 9.90 \\ 1.00 & 0 & 0 & -1.15 & 0 & 0 & 0 \\ 0 & 1.00 & 0 & 0 & -1.15 & 0 & 0 \\ 0 & 0 & 1.00 & 0 & 0 & -1.15 & 0 \\ 0 & 0 & 0 & 0.10 & 0 & 0 & -1.00 \end{bmatrix} \quad A_{W1}=0.01*\begin{bmatrix} -6.93 & 0 & 0 & 0 & 0 & -86.4 & 0 \\ 0 & -6.93 & 0 & -86.4 & 0 & 0 & 0 \\ 0 & 0 & -6.93 & 0 & -0.15 & 0 & 9.90 \\ 1.00 & 0 & 0 & -1.15 & 0 & 0 & 0 \\ 0 & 1.00 & 0 & 0 & -1.15 & 0 & 0 \\ 0 & 0 & 1.00 & 0 & 0 & -1.15 & 0 \\ 0 & 0 & 0 & 0.10 & 0 & 0 & -1.00 \end{bmatrix}$$

$$A_2=\begin{bmatrix} -6.93 & 0 & 0 & 0 & 0 & -86.4 & 0 \\ 0 & -6.93 & 0 & -86.4 & 0 & 0 & 0 \\ 0 & 0 & -6.93 & 0 & -0.15 & 0 & 8.65 \\ 1.00 & 0 & 0 & -1.15 & 0 & 0 & 0 \\ 0 & 1.00 & 0 & 0 & -1.15 & 0 & 0 \\ 0 & 0 & 1.00 & 0 & 0 & -1.15 & 0 \\ 0 & 0 & 0 & 0.10 & 0 & 0 & -1.00 \end{bmatrix} \quad A_{W2}=0.01*\begin{bmatrix} -6.93 & 0 & 0 & 0 & 0 & -86.4 & 0 \\ 0 & -6.93 & 0 & -86.4 & 0 & 0 & 0 \\ 0 & 0 & -6.93 & 0 & -0.15 & 0 & 8.65 \\ 1.00 & 0 & 0 & -1.15 & 0 & 0 & 0 \\ 0 & 1.00 & 0 & 0 & -1.15 & 0 & 0 \\ 0 & 0 & 1.00 & 0 & 0 & -1.15 & 0 \\ 0 & 0 & 0 & 0.10 & 0 & 0 & -1.00 \end{bmatrix}$$

$$A_3=\begin{bmatrix} -6.93 & 0 & 0 & 0 & 0 & -86.4 & 0 \\ 0 & -6.93 & 0 & -86.4 & 0 & 0 & 0 \\ 0 & 0 & -6.93 & 0 & -0.00 & 0 & 9.90 \\ 1.00 & 0 & 0 & -1.15 & 0 & 0 & 0 \\ 0 & 1.00 & 0 & 0 & -1.15 & 0 & 0 \\ 0 & 0 & 1.00 & 0 & 0 & -1.15 & 0 \\ 0 & 0 & 0 & 0.10 & 0 & 0 & -1.00 \end{bmatrix} \quad A_{W3}=0.01*\begin{bmatrix} -6.93 & 0 & 0 & 0 & 0 & -86.4 & 0 \\ 0 & -6.93 & 0 & -86.4 & 0 & 0 & 0 \\ 0 & 0 & -6.93 & 0 & -0.00 & 0 & 9.90 \\ 1.00 & 0 & 0 & -1.15 & 0 & 0 & 0 \\ 0 & 1.00 & 0 & 0 & -1.15 & 0 & 0 \\ 0 & 0 & 1.00 & 0 & 0 & -1.15 & 0 \\ 0 & 0 & 0 & 0.10 & 0 & 0 & -1.00 \end{bmatrix}$$

$$A_4=\begin{bmatrix} -6.93 & 0 & 0 & 0 & 0 & -86.4 & 0 \\ 0 & -6.93 & 0 & -86.4 & 0 & 0 & 0 \\ 0 & 0 & -6.93 & 0 & -0.00 & 0 & 8.65 \\ 1.00 & 0 & 0 & -1.15 & 0 & 0 & 0 \\ 0 & 1.00 & 0 & 0 & -1.15 & 0 & 0 \\ 0 & 0 & 1.00 & 0 & 0 & -1.15 & 0 \\ 0 & 0 & 0 & 0.10 & 0 & 0 & -1.00 \end{bmatrix} \quad A_{W4}=0.01*\begin{bmatrix} -6.93 & 0 & 0 & 0 & 0 & -86.4 & 0 \\ 0 & -6.93 & 0 & -86.4 & 0 & 0 & 0 \\ 0 & 0 & -6.93 & 0 & -0.00 & 0 & 8.65 \\ 1.00 & 0 & 0 & -1.15 & 0 & 0 & 0 \\ 0 & 1.00 & 0 & 0 & -1.15 & 0 & 0 \\ 0 & 0 & 1.00 & 0 & 0 & -1.15 & 0 \\ 0 & 0 & 0 & 0.10 & 0 & 0 & -1.00 \end{bmatrix}$$

$$A_5=\begin{bmatrix} -6.93 & 0 & 0 & 0 & 0 & -86.4 & 0 \\ 0 & -6.93 & 0 & -0.001 & 0 & 0 & 0 \\ 0 & 0 & -6.93 & 0 & -0.15 & 0 & 9.90 \\ 1.00 & 0 & 0 & -1.15 & 0 & 0 & 0 \\ 0 & 1.00 & 0 & 0 & -1.15 & 0 & 0 \\ 0 & 0 & 1.00 & 0 & 0 & -1.15 & 0 \\ 0 & 0 & 0 & 0.10 & 0 & 0 & -1.00 \end{bmatrix} \quad A_{W5}=0.01*\begin{bmatrix} -6.93 & 0 & 0 & 0 & 0 & -86.4 & 0 \\ 0 & -6.93 & 0 & -0.001 & 0 & 0 & 0 \\ 0 & 0 & -6.93 & 0 & -0.15 & 0 & 9.90 \\ 1.00 & 0 & 0 & -1.15 & 0 & 0 & 0 \\ 0 & 1.00 & 0 & 0 & -1.15 & 0 & 0 \\ 0 & 0 & 1.00 & 0 & 0 & -1.15 & 0 \\ 0 & 0 & 0 & 0.10 & 0 & 0 & -1.00 \end{bmatrix}$$

$$A_6 = \begin{bmatrix} -6.93 & 0 & 0 & 0 & 0 & -86.4 & 0 \\ 0 & -6.93 & 0 & -0.001 & 0 & 0 & 0 \\ 0 & 0 & -6.93 & 0 & -0.15 & 0 & 8.65 \\ 1.00 & 0 & 0 & -1.15 & 0 & 0 & 0 \\ 0 & 1.00 & 0 & 0 & -1.15 & 0 & 0 \\ 0 & 0 & 1.00 & 0 & 0 & -1.15 & 0 \\ 0 & 0 & 0 & 0.10 & 0 & 0 & -1.00 \end{bmatrix} \quad A_{W6} = 0.01* \begin{bmatrix} -6.93 & 0 & 0 & 0 & 0 & -86.4 & 0 \\ 0 & -6.93 & 0 & -0.001 & 0 & 0 & 0 \\ 0 & 0 & -6.93 & 0 & -0.15 & 0 & 9.90 \\ 1.00 & 0 & 0 & -1.15 & 0 & 0 & 0 \\ 0 & 1.00 & 0 & 0 & -1.15 & 0 & 0 \\ 0 & 0 & 1.00 & 0 & 0 & -1.15 & 0 \\ 0 & 0 & 0 & 0.10 & 0 & 0 & -1.00 \end{bmatrix}$$

$$A_7 = \begin{bmatrix} -6.93 & 0 & 0 & 0 & 0 & -86.4 & 0 \\ 0 & -6.93 & 0 & -0.001 & 0 & 0 & 0 \\ 0 & 0 & -6.93 & 0 & -0.00 & 0 & 9.90 \\ 1.00 & 0 & 0 & -1.15 & 0 & 0 & 0 \\ 0 & 1.00 & 0 & 0 & -1.15 & 0 & 0 \\ 0 & 0 & 1.00 & 0 & 0 & -1.15 & 0 \\ 0 & 0 & 0 & 0.10 & 0 & 0 & -1.00 \end{bmatrix} \quad A_{W7} = 0.01* \begin{bmatrix} -6.93 & 0 & 0 & 0 & 0 & -86.4 & 0 \\ 0 & -6.93 & 0 & -0.001 & 0 & 0 & 0 \\ 0 & 0 & -6.93 & 0 & -0.00 & 0 & 9.90 \\ 1.00 & 0 & 0 & -1.15 & 0 & 0 & 0 \\ 0 & 1.00 & 0 & 0 & -1.15 & 0 & 0 \\ 0 & 0 & 1.00 & 0 & 0 & -1.15 & 0 \\ 0 & 0 & 0 & 0.10 & 0 & 0 & -1.00 \end{bmatrix}$$

$$A_8 = \begin{bmatrix} -6.93 & 0 & 0 & 0 & 0 & -86.4 & 0 \\ 0 & -6.93 & 0 & -0.001 & 0 & 0 & 0 \\ 0 & 0 & -6.93 & 0 & -0.00 & 0 & 8.65 \\ 1.00 & 0 & 0 & -1.15 & 0 & 0 & 0 \\ 0 & 1.00 & 0 & 0 & -1.15 & 0 & 0 \\ 0 & 0 & 1.00 & 0 & 0 & -1.15 & 0 \\ 0 & 0 & 0 & 0.10 & 0 & 0 & -1.00 \end{bmatrix} \quad A_{W8} = 0.01* \begin{bmatrix} -6.93 & 0 & 0 & 0 & 0 & -86.4 & 0 \\ 0 & -6.93 & 0 & -0.001 & 0 & 0 & 0 \\ 0 & 0 & -6.93 & 0 & -0.00 & 0 & 8.65 \\ 1.00 & 0 & 0 & -1.15 & 0 & 0 & 0 \\ 0 & 1.00 & 0 & 0 & -1.15 & 0 & 0 \\ 0 & 0 & 1.00 & 0 & 0 & -1.15 & 0 \\ 0 & 0 & 0 & 0.10 & 0 & 0 & -1.00 \end{bmatrix}$$

$$A_9 = \begin{bmatrix} -6.93 & 0 & 0 & 0 & 0 & -0.001 & 0 \\ 0 & -6.93 & 0 & -86.4 & 0 & 0 & 0 \\ 0 & 0 & -6.93 & 0 & -0.15 & 0 & 9.90 \\ 1.00 & 0 & 0 & -1.15 & 0 & 0 & 0 \\ 0 & 1.00 & 0 & 0 & -1.15 & 0 & 0 \\ 0 & 0 & 1.00 & 0 & 0 & -1.15 & 0 \\ 0 & 0 & 0 & 0.10 & 0 & 0 & -1.00 \end{bmatrix} \quad A_{W9} = 0.01* \begin{bmatrix} -6.93 & 0 & 0 & 0 & 0 & -0.001 & 0 \\ 0 & -6.93 & 0 & -86.4 & 0 & 0 & 0 \\ 0 & 0 & -6.93 & 0 & -0.15 & 0 & 9.90 \\ 1.00 & 0 & 0 & -1.15 & 0 & 0 & 0 \\ 0 & 1.00 & 0 & 0 & -1.15 & 0 & 0 \\ 0 & 0 & 1.00 & 0 & 0 & -1.15 & 0 \\ 0 & 0 & 0 & 0.10 & 0 & 0 & -1.00 \end{bmatrix}$$

$$A_{10} = \begin{bmatrix} -6.93 & 0 & 0 & 0 & 0 & -0.001 & 0 \\ 0 & -6.93 & 0 & -86.4 & 0 & 0 & 0 \\ 0 & 0 & -6.93 & 0 & -0.15 & 0 & 8.65 \\ 1.00 & 0 & 0 & -1.15 & 0 & 0 & 0 \\ 0 & 1.00 & 0 & 0 & -1.15 & 0 & 0 \\ 0 & 0 & 1.00 & 0 & 0 & -1.15 & 0 \\ 0 & 0 & 0 & 0.10 & 0 & 0 & -1.00 \end{bmatrix} \quad A_{W10} = 0.01* \begin{bmatrix} -6.93 & 0 & 0 & 0 & 0 & -0.001 & 0 \\ 0 & -6.93 & 0 & -86.4 & 0 & 0 & 0 \\ 0 & 0 & -6.93 & 0 & -0.15 & 0 & 8.65 \\ 1.00 & 0 & 0 & -1.15 & 0 & 0 & 0 \\ 0 & 1.00 & 0 & 0 & -1.15 & 0 & 0 \\ 0 & 0 & 1.00 & 0 & 0 & -1.15 & 0 \\ 0 & 0 & 0 & 0.10 & 0 & 0 & -1.00 \end{bmatrix}$$

$$A_{11} = \begin{bmatrix} -6.93 & 0 & 0 & 0 & 0 & -0.001 & 0 \\ 0 & -6.93 & 0 & -86.4 & 0 & 0 & 0 \\ 0 & 0 & -6.93 & 0 & -0.00 & 0 & 9.90 \\ 1.00 & 0 & 0 & -1.15 & 0 & 0 & 0 \\ 0 & 1.00 & 0 & 0 & -1.15 & 0 & 0 \\ 0 & 0 & 1.00 & 0 & 0 & -1.15 & 0 \\ 0 & 0 & 0 & 0.10 & 0 & 0 & -1.00 \end{bmatrix} \quad A_{W11} = 0.01* \begin{bmatrix} -6.93 & 0 & 0 & 0 & 0 & -0.001 & 0 \\ 0 & -6.93 & 0 & -86.4 & 0 & 0 & 0 \\ 0 & 0 & -6.93 & 0 & -0.00 & 0 & 9.90 \\ 1.00 & 0 & 0 & -1.15 & 0 & 0 & 0 \\ 0 & 1.00 & 0 & 0 & -1.15 & 0 & 0 \\ 0 & 0 & 1.00 & 0 & 0 & -1.15 & 0 \\ 0 & 0 & 0 & 0.10 & 0 & 0 & -1.00 \end{bmatrix}$$

$$A_{12} = \begin{bmatrix} -6.93 & 0 & 0 & 0 & 0 & -0.001 & 0 \\ 0 & -6.93 & 0 & -86.4 & 0 & 0 & 0 \\ 0 & 0 & -6.93 & 0 & -0.00 & 0 & 8.65 \\ 1.00 & 0 & 0 & -1.15 & 0 & 0 & 0 \\ 0 & 1.00 & 0 & 0 & -1.15 & 0 & 0 \\ 0 & 0 & 1.00 & 0 & 0 & -1.15 & 0 \\ 0 & 0 & 0 & 0.10 & 0 & 0 & -1.00 \end{bmatrix} \quad A_{W12} = 0.01* \begin{bmatrix} -6.93 & 0 & 0 & 0 & 0 & -0.001 & 0 \\ 0 & -6.93 & 0 & -86.4 & 0 & 0 & 0 \\ 0 & 0 & -6.93 & 0 & -0.00 & 0 & 8.65 \\ 1.00 & 0 & 0 & -1.15 & 0 & 0 & 0 \\ 0 & 1.00 & 0 & 0 & -1.15 & 0 & 0 \\ 0 & 0 & 1.00 & 0 & 0 & -1.15 & 0 \\ 0 & 0 & 0 & 0.10 & 0 & 0 & -1.00 \end{bmatrix}$$

$$A_{13}=\begin{bmatrix} -6.93 & 0 & 0 & 0 & 0 & -0.001 & 0 \\ 0 & -6.93 & 0 & -0.001 & 0 & 0 & 0 \\ 0 & 0 & -6.93 & 0 & -0.15 & 0 & 9.90 \\ 1.00 & 0 & 0 & -1.15 & 0 & 0 & 0 \\ 0 & 1.00 & 0 & 0 & -1.15 & 0 & 0 \\ 0 & 0 & 1.00 & 0 & 0 & -1.15 & 0 \\ 0 & 0 & 0 & 0.10 & 0 & 0 & -1.00 \end{bmatrix} \quad A_{W13}=0.01*\begin{bmatrix} -6.93 & 0 & 0 & 0 & 0 & -0.001 & 0 \\ 0 & -6.93 & 0 & -0.001 & 0 & 0 & 0 \\ 0 & 0 & -6.93 & 0 & -0.15 & 0 & 9.90 \\ 1.00 & 0 & 0 & -1.15 & 0 & 0 & 0 \\ 0 & 1.00 & 0 & 0 & -1.15 & 0 & 0 \\ 0 & 0 & 1.00 & 0 & 0 & -1.15 & 0 \\ 0 & 0 & 0 & 0.10 & 0 & 0 & -1.00 \end{bmatrix}$$

$$A_{14}=\begin{bmatrix} -6.93 & 0 & 0 & 0 & 0 & -0.001 & 0 \\ 0 & -6.93 & 0 & -0.001 & 0 & 0 & 0 \\ 0 & 0 & -6.93 & 0 & -0.15 & 0 & 8.65 \\ 1.00 & 0 & 0 & -1.15 & 0 & 0 & 0 \\ 0 & 1.00 & 0 & 0 & -1.15 & 0 & 0 \\ 0 & 0 & 1.00 & 0 & 0 & -1.15 & 0 \\ 0 & 0 & 0 & 0.10 & 0 & 0 & -1.00 \end{bmatrix} \quad A_{W14}=0.01*\begin{bmatrix} -6.93 & 0 & 0 & 0 & 0 & -0.001 & 0 \\ 0 & -6.93 & 0 & -0.001 & 0 & 0 & 0 \\ 0 & 0 & -6.93 & 0 & -0.15 & 0 & 8.65 \\ 1.00 & 0 & 0 & -1.15 & 0 & 0 & 0 \\ 0 & 1.00 & 0 & 0 & -1.15 & 0 & 0 \\ 0 & 0 & 1.00 & 0 & 0 & -1.15 & 0 \\ 0 & 0 & 0 & 0.10 & 0 & 0 & -1.00 \end{bmatrix}$$

$$A_{15}=\begin{bmatrix} -6.93 & 0 & 0 & 0 & 0 & -0.001 & 0 \\ 0 & -6.93 & 0 & -0.001 & 0 & 0 & 0 \\ 0 & 0 & -6.93 & 0 & -0.00 & 0 & 9.90 \\ 1.00 & 0 & 0 & -1.15 & 0 & 0 & 0 \\ 0 & 1.00 & 0 & 0 & -1.15 & 0 & 0 \\ 0 & 0 & 1.00 & 0 & 0 & -1.15 & 0 \\ 0 & 0 & 0 & 0.10 & 0 & 0 & -1.00 \end{bmatrix} \quad A_{W15}=0.01*\begin{bmatrix} -6.93 & 0 & 0 & 0 & 0 & -0.001 & 0 \\ 0 & -6.93 & 0 & -0.001 & 0 & 0 & 0 \\ 0 & 0 & -6.93 & 0 & -0.00 & 0 & 9.90 \\ 1.00 & 0 & 0 & -1.15 & 0 & 0 & 0 \\ 0 & 1.00 & 0 & 0 & -1.15 & 0 & 0 \\ 0 & 0 & 1.00 & 0 & 0 & -1.15 & 0 \\ 0 & 0 & 0 & 0.10 & 0 & 0 & -1.00 \end{bmatrix}$$

$$A_{16}=\begin{bmatrix} -6.93 & 0 & 0 & 0 & 0 & -0.001 & 0 \\ 0 & -6.93 & 0 & -0.001 & 0 & 0 & 0 \\ 0 & 0 & -6.93 & 0 & -0.00 & 0 & 8.65 \\ 1.00 & 0 & 0 & -1.15 & 0 & 0 & 0 \\ 0 & 1.00 & 0 & 0 & -1.15 & 0 & 0 \\ 0 & 0 & 1.00 & 0 & 0 & -1.15 & 0 \\ 0 & 0 & 0 & 0.10 & 0 & 0 & -1.00 \end{bmatrix} \quad A_{W16}=0.01*\begin{bmatrix} -6.93 & 0 & 0 & 0 & 0 & -0.001 & 0 \\ 0 & -6.93 & 0 & -0.001 & 0 & 0 & 0 \\ 0 & 0 & -6.93 & 0 & -0.00 & 0 & 8.65 \\ 1.00 & 0 & 0 & -1.15 & 0 & 0 & 0 \\ 0 & 1.00 & 0 & 0 & -1.15 & 0 & 0 \\ 0 & 0 & 1.00 & 0 & 0 & -1.15 & 0 \\ 0 & 0 & 0 & 0.10 & 0 & 0 & -1.00 \end{bmatrix}$$
